# Lightweight interactive feature inference network for single-image super-resolution

**DOI:** 10.1038/s41598-024-62633-8

**Published:** 2024-05-21

**Authors:** Li Wang, Xing Li, Wei Tian, Jianhua Peng, Rui Chen

**Affiliations:** 1 School of Computer and Software, Nanjing Vocational University of Industry Technology, Nanjing, 210023 China; 2 College of Information Science and Technology, College of Artificial Intelligence, Nanjing Forestry University, Nanjing, 210037 China; 3 College of Computer and Information Engineering, Guizhou University of Commerce, Guiyang, 550014 China

**Keywords:** Super-resolution, Convolution neural network, Transformer, Local and global priors, Engineering, Electrical and electronic engineering

## Abstract

The emergence of convolutional neural network (CNN) and transformer has recently facilitated significant advances in image super-resolution (SR) tasks. However, these networks commonly construct complex structures, having huge model parameters and high computational costs, to boost reconstruction performance. In addition, they do not consider the structural prior well, which is not conducive to high-quality image reconstruction. In this work, we devise a lightweight interactive feature inference network (IFIN), complementing the strengths of CNN and Transformer, for effective image SR reconstruction. Specifically, the interactive feature aggregation module (IFAM), implemented by structure-aware attention block (SAAB), Swin Transformer block (SWTB), and enhanced spatial adaptive block (ESAB), serves as the network backbone, progressively extracts more dedicated features to facilitate the reconstruction of high-frequency details in the image. SAAB adaptively recalibrates local salient structural information, and SWTB effectively captures rich global information. Further, ESAB synergetically complements local and global priors to ensure the consistent fusion of diverse features, achieving high-quality reconstruction of images. Comprehensive experiments reveal that our proposed networks attain state-of-the-art reconstruction accuracy on benchmark datasets while maintaining low computational demands. Our code and results are available at: https://github.com/wwaannggllii/IFIN.

## Introduction

Image super-resolution (SR) involves the process of restoring a high-resolution (HR) image from a corresponding degraded low-resolution (LR) image. Currently, image SR is favored for its broad applications in medical imaging, surveillance, and face recognition. The ill-posed problem of SR, however, presents a challenge where multiple HR images can be reconstructed from a single LR image. Many SR methods have been introduced to tackle this issue, such as interpolation-based, reconstruction-based, and learning-based methods.

The emergence of convolutional neural networks (CNN) recently instigated a profound revolution in SR tasks. From SRCNN^1^ (incorporating only three convolutional layers) to RCAN^2^ (encompassing over 400 layers), there has been a consistent increase in network depth, width, and complexity. This development trend benefits network representation capabilities while achieving reconstruction performance improvements. For example, enhanced deep SR network (EDSR)^3^ and non-local sparse attention (NLSA)^4^ with parameters of 43M and 42M, respectively, can produce significant restoration effects by powerful nonlinear learning. Nevertheless, the practical application of the majority of these CNN-based methods in real-world scenarios remains challenging, primarily owing to their demanding memory and computational requirements. Despite various efforts directed toward reducing the number of network parameters and operations, most methods struggle to maintain good reconstruction performance. A deeply recursive convolutional network(DRCN)^5^ has fewer model parameters by recursive paradigm, but the reconstruction accuracy is lower. Cascading residual network (CARN)^6^ implemented a cascading residual architecture by introducing a cascading mechanism, where the whole structure is lightweight but poorly performing. To better balance network performance and computational cost, researchers have introduced attention mechanisms into the SR task. LatticeNet^7^, DRSAN^8^, and A2F^9^ exploited different attention mechanisms to focus on informative features, boosting reconstruction performance while maintaining moderate computational demands. Notably, most existing attention mechanisms lack structural priors, which are crucial for image detail recovery. Therefore, it is essential to devise an effective and lightweight network that explores structural information within attention mechanisms for reconstructing high-quality HR images.

Conversely, CNN-based SR approaches struggle to address global dependencies, primarily attributed to the inherent local properties of convolutional operations. As an alternative to CNN, Transformer can capture global interactions between contexts through the self-attention mechanism, making them widely adopted in the field of SR. Swin Transformer^10^ has exhibited significant promise by harnessing the advantages of both CNN and Transformer. Later, the hybrid structure of CNN and Transformer has gradually become a mainstream trend in research. An efficient long-range attention network (ELAN)^11^ proposed a share attention technique to speed up the calculation in its group multi-head self-attention. Hybrid network of CNN and Transformer (HNCT)^12^ combined CNN and Transformer to extract deep features in consideration of both local and non-local priors. Similarly, cross-receptive focused inference network (CFIN)^13^ elegantly integrated CNN and Transformer and achieved competitive performance. In aggregate enriched features extracted from both CNN and Transformer (ACT)^14^, it exploited multi-scale local and non-local attributes to improve SR quality. Benefiting from the advantages of this hybrid architecture, we further explore the processing of local and global information to obtain more valuable information for HR reconstruction.

**Figure 1:**
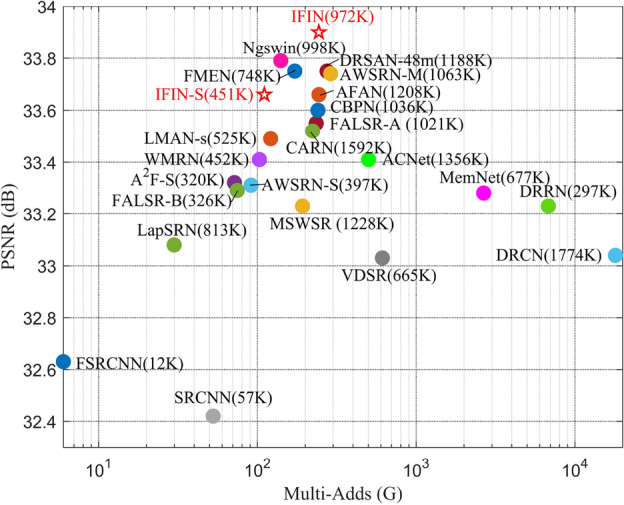
PSNR and model size comparison of our methods (red star) with mainstream SR networks on Set14 for scale factor $\times$2.

In this study, a lightweight interactive feature inference network (IFIN) is implemented for image SR tasks. Specifically, a series of interactive feature aggregation modules (IFAM) capture more abstract depth features in a coarse-to-fine fashion. IFAM is supported by structure-aware attention block (SAAB), Swin Transformer block (SWTB), and enhanced spatial adaptive block (ESAB), complementing and integrating different features synergistically. SAAB and SWTB extract local structure and global aware priors, respectively. These two different feature properties are merged and fused in ESAB, recovering natural and realistic textures of HR images. As reported in Fig. 1, our proposed networks deliver a favorable trade-off between performance and model size, outperforming most renowned SR models.

In brief, we make three primary contributions.

1. We introduce a lightweight and efficient model, dubbed IFIN, which utilizes chain-stacked IFAMs to extrapolate image features from coarse to fine granularity. Supported by SAAB, SWTB, and ESAB, the IFAM effectively leverages both local and global a priori knowledge, thereby enhancing the network’s discriminatory capabilities. IFIN achieves favorable performance with modest computing requirements, surpassing most well-known lightweight approaches.
2. We propose SAAB, which incorporates asymmetrical convolution within the attention mechanism. This integration facilitates the learning of intricate structural information and the generation of more generalized weights, effectively emphasizing critical target regions.
3. We propose ESAB, which synergistically aggregates local structural information from SAAB and global aware information from SWTB. In such a way, ESAB can enhance the network’s adaptability to various image contents and scenes, thereby significantly improving image reconstruction performance.

## Related work

### CNN-based image SR

Dong et al.^1^ were pioneers in applying CNN to the SR domain, developing the SRCNN model which outperformed traditional methods in achieving superior SR results. Inspired by this idea, Kim et al.^15^ increased the network depth to 20 layers and further improved the reconstruction performance. Later, a wide variety of CNN-based SR network designs emerged to facilitate reconstruction accuracy, such as increasing network depth, expanding network width, and designing complex network architectures. For instance, enhanced deep SR network (EDSR)^3^, residual dense network (RDN)^16^, holistic attention network (HAN)^17^, and dual interactive implicit neural network (DIINN)^18^ were very deep networks that had very dominant restoration accuracy, but they suffered from a very large number of parameters and computations. Instead of designing huge networks, efficient SR methods provide a good balance of performance and model capacity. CARN^6^ leveraged group convolution and a cascade scheme to decrease model capacity and enhance network representation. IDN^19^ distilled more useful information for SR reconstruction via distillation technology to reduce network parameters. LatticeNet^7^ designed lattice blocks that favor the lightweight SR framework, reducing the number of parameters by about half while maintaining similar SR performance. Additionally, to promote the efficiency of feature utilization, several works have incorporated the attention mechanism into the SR field. MemNet^20^ and channel-wise and spatial feature modulation (CSFM)^21^ aggregated channel attention and spatial attention, exploring the interdependencies between channel and spatial attributes. Plus, PAN^22^ and DRSAN^8^ conducted attention mechanisms that adaptively rescaled features using three-dimensional (3D) attention maps, resulting in improved SR outcomes. Although different solutions can generate different lightweight SR results, they ignore the exploration and use of structural priors which are beneficial for image detail reconstruction.

### Transformer-based image SR

As an alternative to CNN, Transformer which adopts the self-attention mechanism has escalated the accuracy of various computer vision tasks. One pioneering work is Vision Transformer (ViT )^23^, which flattened two-dimensional (2D) image patches in a vector and delivered them into the Transformer structure, obtaining remarkable performance gains. Shortly afterward, an increasing number of Transformer-based approaches have sprung up in SR tasks. Image processing Transformer (IPT)^24^, based on ViT, acquired better restoration results in denoising, deraining, and SR tasks. Instead of the standard self-attention, Swin Transformer^10^ adopted the Swin Transformer block by incorporating convolutional layers within the block to enforce local connectivity. Currently, the popular direction of research in the SR domain is the hybrid structure of CNN and Transformer. Many research efforts have demonstrated the effectiveness of this hybrid architecture, mainly thanks to the fact that CNN structure can extract local features while the Transformer structure can establish global features. For instance, efficient super-resolution Transformer (ESRT)^25^, ELAN^11^, hierarchical patch Transformer (HIPA)^26^, and ACT^27^ extracted and enhanced feature representations by hybridizing CNN backbone and Transformer backbone, acquiring better performance than most Transformer-based and CNN-based methods. Indeed, effectively incorporating both local features and global information into lightweight networks is crucial for achieving high-performance results. In this study, we aim to enhance the flexibility and robustness of local structure and global feature priors, thereby achieving HR image restoration.

## Proposed method

### Overall network architecture

**Figure 2:**
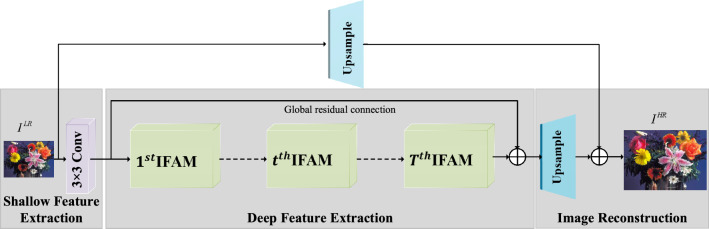
The architecture of our proposed IFIN, which consists of *T* IFAMs to gradually infer rich contextual features.

In this work, we construct a lightweight interactive feature inference network (IFIN) for image SR fields. As depicted in Fig. 2, the entire workflow of IFIN consists of a shallow feature extraction module, several interactive feature aggregation modules (IFAM), and an upsample part. Firstly, the LR image passes through a 3 $\times$ 3 convolution to distill shallow features, which can be defined as:

1 $$\begin{aligned} {F_0} = {H_{SFE}}\left( {{I^{LR}}} \right) \end{aligned}$$

where ${I^{LR}} \in {{\mathbb {R}}^{H \times W \times 3}}$ denotes the LR input images. *H* and *W* indicate the height and width of the image. ${H_{SFE}}( \cdot )$ is the 3$\times$3 convolution operation and ${F_0} \in {{\mathbb {R}}^{H \times W \times C}}$ is the extracted shallow features, where *C* is the number of channels. Then, ${F_0}$ will be transmitted to *T* chained stacking IFAMs for learning more abstract high-level features. IFAM is composed of a structure-aware attention block (SAAB), Swin Transformer block (SWTB), and enhanced spatial adaptive block (ESAB), which will be described in “Interactive feature aggregation module (IFAM)” . The process can be expressed as follows:

2 $$\begin{aligned} F_{_{IFAM}}^t = \mathrm{{\;}}H_{\mathrm{{IFAM}}}^t\left( {{F_{t - 1}}} \right) = H_{\mathrm{{IFAM}}}^t\left( {H_{\mathrm{{IFAM}}}^{t - 1}\left( { \cdots H_{\mathrm{{IFAM}}}^1\left( {{F_0}} \right) \cdots } \right) } \right) \end{aligned}$$

where $\mathrm{{\;}}H_{\mathrm{{IFAM}}}^t( \cdot )$ indicates the operation of the t-th IFAM. ${F_{_{IFAM}}^{t - 1}} \in {{\mathbb {R}}^{H \times W \times C}}$ and ${F_{_{IFAM}}^t} \in {{\mathbb {R}}^{H \times W \times C}}$ are the input and output feature maps of the *t*-th IFAM. Finally, the extracted deeper features ${F_{_{IFAM}}^T \in {{\mathbb {R}}^{H \times W \times C}}}$ is upsampled to the ideal HR image size, which can be expressed as:

3 $$\begin{aligned} {I^{HR}} = {H_{HU}}({F_{_{IFAM}}^T}) + {H_{LU}}({I^{LR}}) \end{aligned}$$

where ${I^{HR}} \in {{\mathbb {R}}^{rH \times rW \times 3}}$ is the HR image, where *r* is the scale factor. ${H_{HU}}(\cdot )$ and ${H_{LU}}(\cdot )$ denote the upsampling operations for the deeper features and input LR image, respectively. Similar to work^28^, both operations integrate a 3 $\times$ 3 convolution for ${H_{HU}}(\cdot )$ and 5$\times$5 convolution for ${H_{LU}}(\cdot )$, as well as a sub-pixel convolutional layer. With this technique, the stability of network training is improved.

We utilize ${L_1}$ norm as the objective function of the proposed IFIN. Assuming a training dataset $\{ I_i^{LR},I_i^{SR}\} _{i = 1}^N$, where $I_i^{LR} \in {{\mathbb {R}}^{H \times W \times 3}}$ and $I_i^{SR} \in {{\mathbb {R}}^{rH \times rW \times 3}}$ denote the *i*-th LR image and the corresponding ground-truth image, respectively. A powerful non-linear mapping function ${H_{IFIN}}(\cdot )$ , capturing the relationship $I_i^{LR}$ and $I_i^{SR}$ using the $L_1^{}$ norm, can be defined as:

4 $$\begin{aligned} \begin{aligned} L\left( \mathrm{{\Theta }} \right)&= \frac{1}{N}\mathop \sum \limits _{i = 1}^N {\left\| {I_i^{SR} - {H_{IFIN}}\left( {I_i^{LR}} \right) } \right\| _1} \\&= \frac{1}{N}\mathop \sum \limits _{i = 1}^N {\left\| {I_i^{SR} - I_i^{HR}} \right\| _1} \end{aligned} \end{aligned}$$

where $\Theta$ is the learnable parameter set of IFIN.

### Interactive feature aggregation module (IFAM)

As the backbone of IFIN, IFAM allows collaborative exploration of the local and global prior of the image, helping to reconstruct a more texture-rich HR image. IFAM is made up of SAAB, SWTB, and ESAB, which are described as follows.

#### Structure-aware attention block (SAAB)

Asymmetric convolution explores structural information by leveraging the vertical and horizontal gradient information parallelly, not only reducing model operations but also helping to recover high-quality images. For instance, Tian et al.^29^ introduced ACNet, which utilizes asymmetric blocks with higher efficiency and fewer parameters. Analogously, Xu et al.^30^ proposed asymmetric attention convolution (AAConv) to gradually extract advanced spatial patterns and spectral features. Considering the excellent structural prior of asymmetric convolution, we embed it into the attention mechanism to focus on more important structural features and improve network representation. Therefore, we propose a structure-aware attention block (SAAB), which embeds asymmetric convolution within the attention path and modulates it with the convolutional path to acquire rich structure-aware features. In contrast to AAConv, we advocate for leveraging structural priors to empower the attention path in learning more generalized weights, followed by adaptive reweighting of the convolutional path to emphasize essential target structural information.

**Figure 3:**
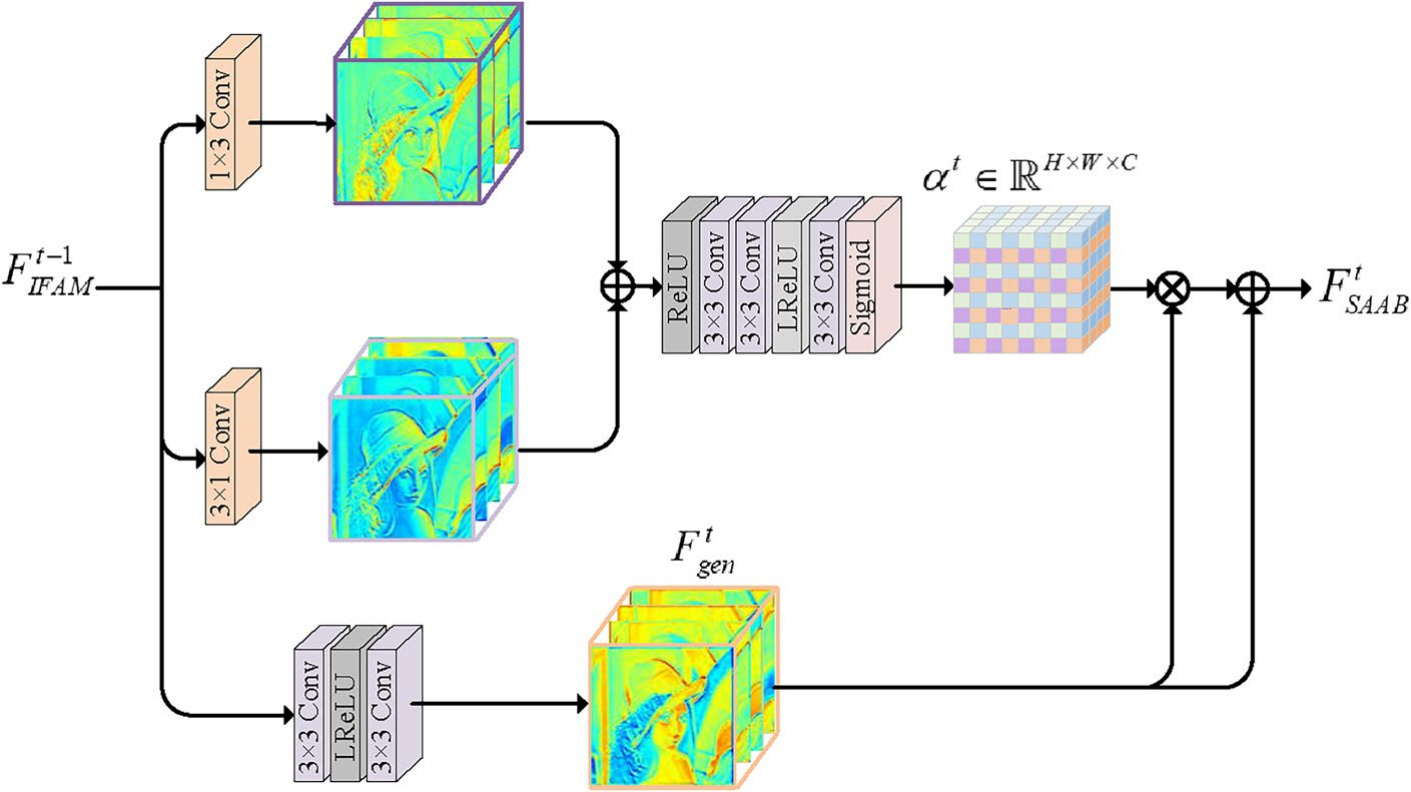
The architecture of SAAB that concentrates on rich structural-aware features.

As shown in Fig.  3, SAAB starts with 1 $\times$ 3 and 3 $\times$ 1 convolutions for structural information exploration, and then passes to three 3 $\times$ 3 convolutions for feature learning, followed by sigmoid to generate 3D modulation coefficients ${\alpha ^t} \in {{\mathbb {R}}^{H \times W \times C}}$. Additionally, to gather more important generalized features $F_{gen}^t \in {{\mathbb {R}}^{H \times W \times C}}$, we use two 3 $\times$ 3 convolutions that are independent of the attention path. Finally, the generalized features are recalibrated by 3D modulation coefficients, acquiring rich discriminative feature representation $F_{SAAB}^t \in {{\mathbb {R}}^{H \times W \times C}}$ for accurate SR reconstruction. The above process can be formulated as follows:

5 $$\begin{aligned} {\alpha ^t} = \sigma \left( {{H_{3 \times 3}}({f_{1 \times 3}}(F_{IFAM}^{t - 1}) + {f_{3 \times 1}}(F_{IFAM}^{t - 1}))} \right) \end{aligned}$$

6 $$\begin{aligned} F_{gen}^t = {f_{3 \times 3}}({f_{3 \times 3}}(F_{IFAM}^{t - 1})) \end{aligned}$$

7 $$\begin{aligned} F_{SAAB}^t = {\alpha ^t} \cdot F_{gen}^t + F_{gen}^t \end{aligned}$$

where $\sigma \left( \cdot \right)$ denotes the sigmoid function. $H\left( \cdot \right)$ and $f\left( \cdot \right)$ are different convolution operations, where the subscripts indicate the sizes of convolution.

#### Swin transformer block (SWTB)

SWTB is derived from the literature^31^ that introduces local attention and shifted window mechanisms to decrease model complexity and achieve efficient learning. We adopt SWTB to learn global context information, acquiring more valuable information to realize detail restoration.

**Figure 4:**
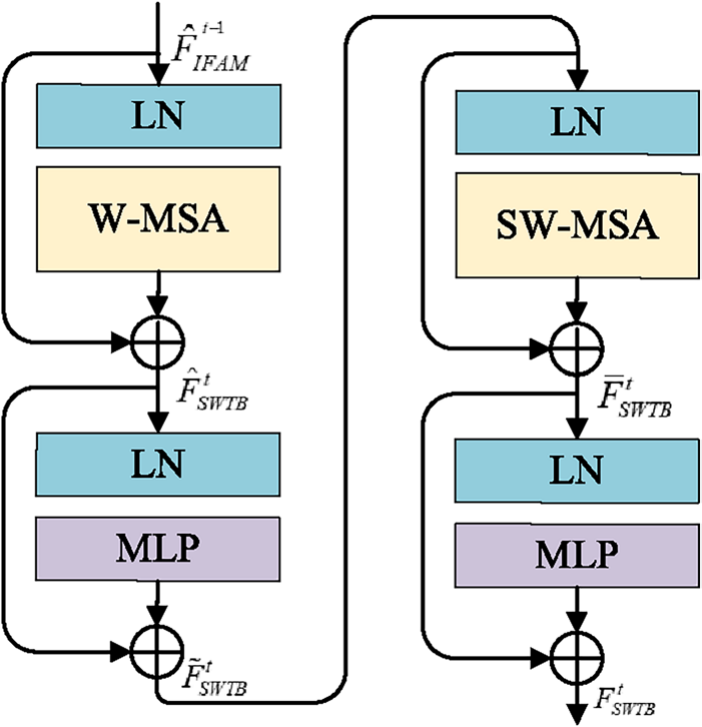
The architecture of SWTB that can model global information effectively.

Figure 4 presents the structure of two consecutive SWTB, containing a LayerNorm (LN) layer, a multi-head self-attention block, residual connection, and two multi-layer perceptrons (MLP). The window-based multi-head self-attention (W-MSA) unit and the shifted window-based multi-head self-attention (SW-MSA) unit are employed in the two successive Transformer blocks, respectively. $F_{IFAM}^{^{t - 1}} \in {^{H \times W \times C}}$ will be linearly projected and reshaped into ${\hat{F}}_{IFAM}^{^{t - 1}} \in {{\mathbb {R}}^{N \times C}}$, where $N = H \times W$. The input feature will be separated into non-overlapping windows, with each window containing M$\times$M patches (set to 8 by default). With the window partitioning mechanism, the continuous SWTB can be represented as:

8 $$\begin{aligned} {\hat{F}}_{SWTB}^t = W{\text{- }}MSA\left( {LN\left( \hat{F_{IFAM}^{^{t - 1}}} \right) } \right) + {\hat{F}}_{IFAM}^{^{t - 1}} \end{aligned}$$

9 $$\begin{aligned} {\tilde{F}}_{SWTB}^t = MLP\left( {LN\left( {{\hat{F}}_{SWTB}^t} \right) } \right) + {\hat{F}}_{SWTB}^t \end{aligned}$$

10 $$\begin{aligned} {\bar{F}}_{SWTB}^t = SW{\text{- }}MSA\left( {LN\left( {\tilde{F}_{SWTB}^t} \right) } \right) + {\tilde{F}}_{SWTB}^t \end{aligned}$$

11 $$\begin{aligned} F_{SWTB}^t = MLP\left( {LN\left( {{\bar{F}}_{SWTB}^t} \right) } \right) + {\bar{F}}_{SWTB}^t \end{aligned}$$

where ${\hat{F}}_{SWTB}^t$, ${\bar{F}}_{SWTB}^t$, ${\tilde{F}}_{SWTB}^t$, and $F_{SWTB}^t$ are the outputs of the (S)W-MSA module and the MLP of the *t*-th block, respectively. The self-attention figured in W-MSA and SW-MSA can be summarized by the following formula:

12 $$\begin{aligned} A\mathrm{{ttention}}\left( {Q,K,V} \right) \mathrm{{ = }} = \mathrm{{softmax}}\left( {\frac{{Q{K^T}}}{{\sqrt{d} }} + B} \right) \mathrm{{V}} \end{aligned}$$

where *Q*, *K*, $V \in {{\mathbb {R}}^{{M^2} \times d}}$ indicate querie, key, and value matrices, respectively. *d* indicates size of the query and key. $B \in {{\mathbb {R}}^{{M^2} \times {M^2}}}$ indicates the relative position bias.

#### Enhanced spatial adaptive block (ESAB)

It is recognized that both the local and global priors of an image contribute to the reconstruction of rich texture details. While we have independently considered the local and non-local features of images from SAAB and SWTB, there is room for further exploration to enhance the flexibility of fusion. By exploiting local and global priors, the network becomes more robust to changes in the input image and is better able to handle different image contents and scenes, favouring the recovery of richer high-frequency information.

**Figure 5:**
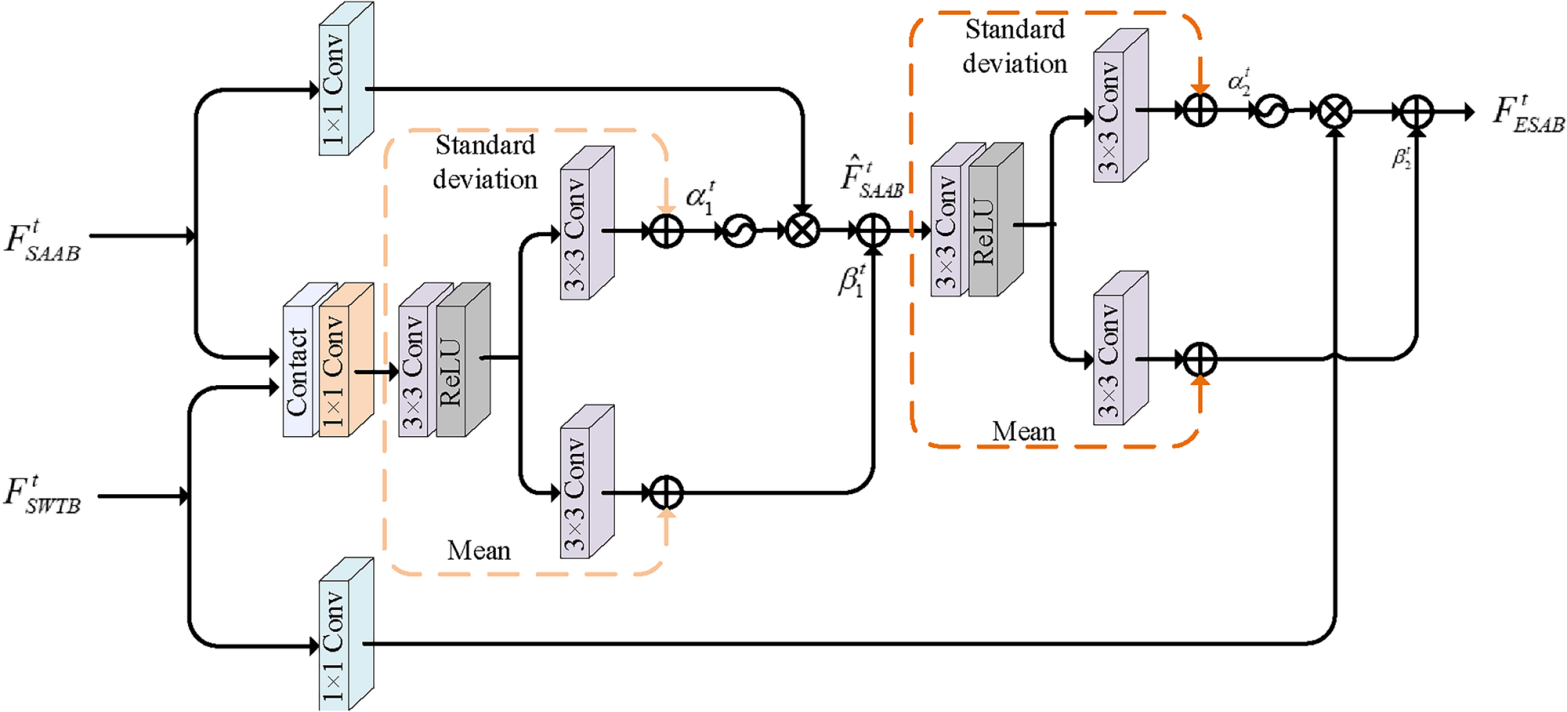
The architecture of ESAB that autonomously aggregates local structure and global feature priors.

Figure 5 illustrates the structure of ESAB. Firstly, the output features $F_{SAAB}^t$ and $F_{\mathrm{{SWTB}}}^t$ are concatenated and processed by 1$\times$1 convolutional layer to harvest diverse types of fused characteristics. Then, 3$\times$3 convolutional layer is exploited to generate the modulation parameters ${\alpha _1^t}$ and ${\beta _1^t}$, which will be updated with the mean and standard deviation of fused characteristics. Subsequently, a sigmoid operation is applied to yield modulation coefficients. Finally, the spatial features $F_{_{SAAB}}^t$ enhanced by a 1$\times$1 convolution are multiplied with modulation coefficients and then added with ${\beta _1^t}$, to acquire spatial modulation features ${{\hat{F}}_{SAAB}^t} \in {{\mathbb {R}}^{H \times W \times C}}$.

13 $$\begin{aligned} {\hat{F}}_{SAAB}^t = {f_{1 \times 1}}\left( {F_{SAAB}^t} \right) \cdot \sigma \left( {{\alpha _1^t}} \right) + {\beta _1^t} \end{aligned}$$

Analogously, the modulated features ${{\hat{F}}_{SAAB}^t}$ are convolved to produce another modulation parameters ${\alpha _2^t}$ and ${\beta _2^t}$, and whose are multiplied and added to the enhanced global features ${\hat{F}}_{\mathrm{{SWTB}}}^t$, distilling global modulation features $F_{\mathrm{{SWTB}}}^t$, which is also the output features $F_{_{IFAM}}^t$ of the *t*-th IFAM.

14 $$\begin{aligned} F_{_{IFAM}}^t = F_{ESAB}^t = {f_{1 \times 1}}\left( {{\hat{F}}_{SWTB}^t} \right) \cdot \sigma \left( {{\alpha _2^t}} \right) + {\beta _2^t} \end{aligned}$$

where $\sigma \left( \cdot \right)$ denotes the sigmoid function. ${f_{1 \times 1}}\left( \cdot \right)$ denoete 1$\times$1 convolution.

## Experiments

### Datasets and metrics

IFIN-S and IFIN are trained on DIV2K^32^ dataset, in which 800 high-quality images are available. Then we test on five benchmark datasets: Set5^33^, Set14^34^, B100^35^, Urban100^36^, and Manga109^37^. Besides, three degradation models, known as bicubic (BI), blur-downscale (BD), and downscale-noise (DN), are leveraged to demonstrate the effectiveness of IFIN. The experimental outcomes are estimated utilizing two metrics, the peak signal-to-noise ratio (PSNR) and structure similarity index (SSIM), on the Y channel of the transformed YCbCr color space.

### Implementation details

In order to build a lightweight architecture, we devise two network variants, referred to as IFIN-S and IFIN. The channels of *C* are configured to 50. We construct IFIN-S by stacking three IFAMs and configuring the group size of the 3 $\times$ 3 convolutions in SAAB to 2. For IFIN, we stack five IFAMs.

Following the BI degradation model, we downsampled the datasets by scale factors of $\times$2, $\times$3, and $\times$4 to produce the corresponding LR images. For the BD and DN degradation models, however, we specifically process the dataset using a scale factor of $\times$3. Each mini-batch comprises 16 image patches, each of size 60 $\times$ 60. To augment the database, both the LR and HR image patches undergo random horizontal flipping as well as rotations of 90$^\circ$, 180$^\circ$, and 270$^\circ$. Before inputting the mini-batch into the model, we normalize it by subtracting the average RGB value calculated from the entire training dataset. The ${L_1}$ paradigm is minimized by employing the Adam optimizer, whose parameters are given as $\beta _1 = 0.9$, $\beta _2 = 0.999$, and $\varepsilon = 10^{-8}$. The initial learning rate is set to 1e−3 at the beginning and is halved every 500 epochs. The detailed hyperparameters utilized for our network architecture are listed in Table 1. Our IFIN-S and IFIN are implemented by exploiting the PyTorch framework on an NVIDIA TESLA V100 GPU.

**Table 1:** Values of hyperparameters for our network.

Hyperparameters	Value
Use_hflip	True
Use_rot	True
Use_shuffle	True
Optim_g	Adam
lr	0.001
Weight_decay	0
betas	[0.9, 0.99]
gamma	0.5
Loss type	L1
Loss_weight	1.0

### Ablation study

In this section, we implement ablation studies to demonstrate the effectiveness of various components of IFIN in enhancing reconstruction accuracy. We respectively get rid of SAAB, ESAB, and SWTB, thus obtaining three additional models. Table 2 reports model capacity, PSNR/SSIM, and time consumption for four models on five benchmark datasets. The time consumption tests are performed on an NVIDIA GeForce RTX 3060 GPU, with results averaged across the datasets. Additionally, we explore the impact of varying the number of IFAMs on network performance, aiming to identify the optimal balance between computational efficiency and enhancement efficacy.

**Table 2:** Ablation studies on effects of SAAB, SWTB, and ESAB.

Model	Params	Multi-Adds	Set5 PSNR(dB)/SSIM	Set14 PSNR(dB)/SSIM	B100 PSNR(dB)/SSIM	Urban100 PSNR(dB)/SSIM	Manga109 PSNR(dB)/SSIM	Time (s)
IFIN w/o SAAB	609K	53.9G	32.26/0.8956	28.66/0.7831	27.60/0.7379	26.14/0.7883	30.62/0.9088	0.08325
IFIN w/o SWTB	875K	60.9G	32.16/0.8947	28.55/0.7814	27.54/0.7358	25.96/0.7854	30.58/0.9061	0.04293
IFIN w/o ESAB	909K	51.7G	32.28/0.8960	28.67/0.7835	27.62/0.7380	26.20/0.7900	30.73/0.9118	0.08046
IFIN	991K	64.6G	32.34/0.8965	28.70/0.7838	27.64/0.7384	26.26/0.7910	30.80/0.9123	0.08921

The average PSNR/SSIM and time consumption on benchmark datasets for scale factor $\times$4.

1. Investigation of SAAB. Our proposed SAAB inherits the property of asymmetric convolution and can learn the structural features of the image for better detail restoration. As reported in Table 2, it is evident that IFIN equipped with SAAB acquires a substantial performance improvement, especially on the structurally complex Urban100 and Manga109 datasets, where SSIM respectively improves by 0.0027 and 0.0035. Despite the fact that the addition of SAAB increases the parameter count by 328K, the performance improvement is significant. Additionally, the increase in time consumption is only 7%. As expected, SAAB facilitates the recovery of high-quality images by embedding structural priors. Figure 6 is the visual heatmap of different stages of IFAM, produced by IFIN w/o SAAB and IFIN, respectively. As for IFIN enabled by SAAB (Fig. 6f–j), obviously, it can effectively outline clear and sharp edge information, validating the ability to explore structural textures. While IFIN w/o SAAB (Fig. 6a–e) not only has low reconstruction accuracy but also displays blurred and distorted structural information.

**Figure 6:**
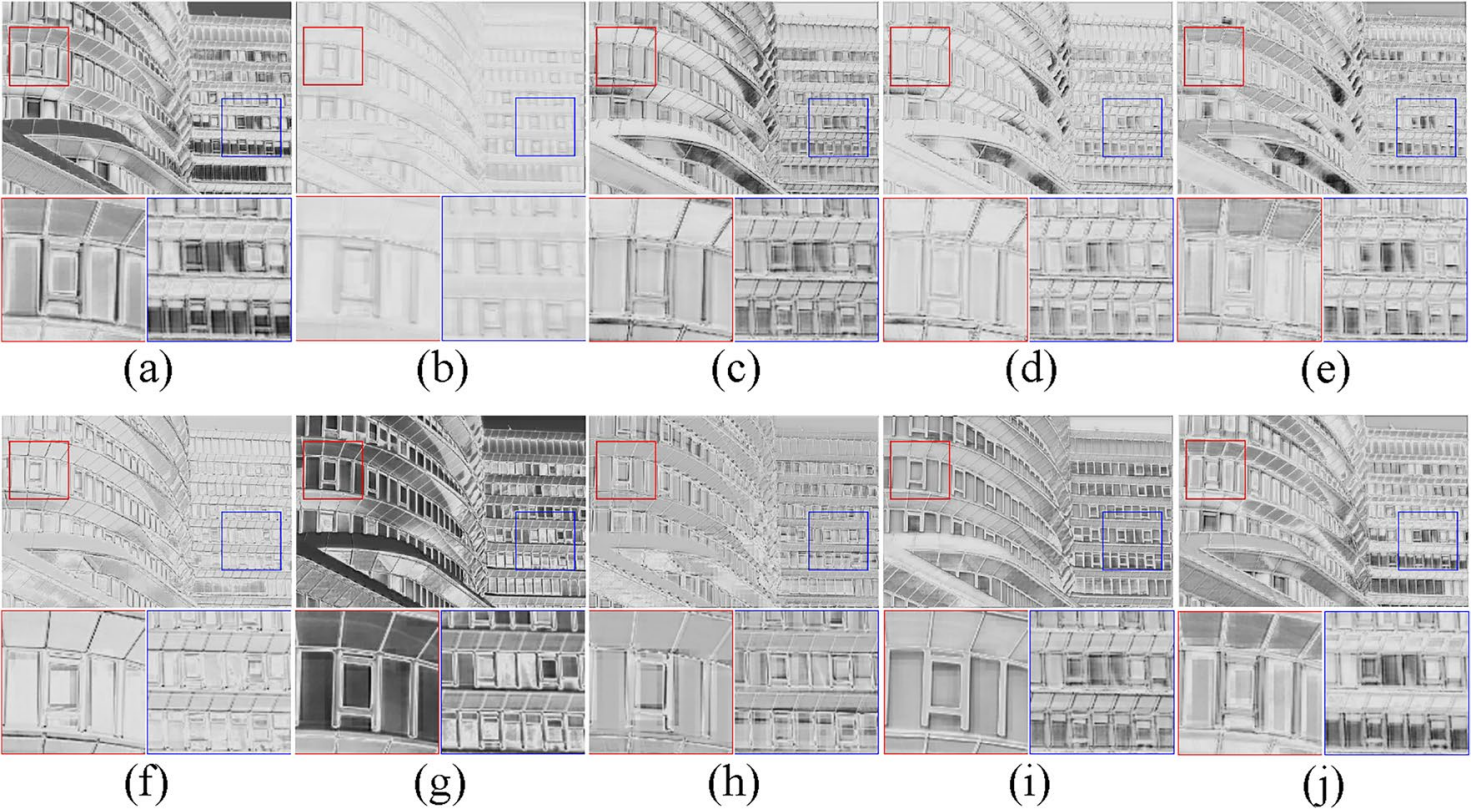
Visualized feature maps of IFIN with and without SAAB. (**a**–**e**) Heatmaps of IFIN without SAAB, (**f**–**j**) show heatmaps of IFIN with SAAB.
2. Investigation of SWTB. SWTB has a strong representation ability and global information utilization, thus facilitating the recovery of more useful characteristics. As depicted in Table 2, by introducing SWTB, the reconstruction results provide at least 0.10 dB gains, which indicates the importance of global dependence in image restoration. The convergence analysis is presented in Fig. 7. We can find that IFIN w/o SWTB converges faster, while the other models are relatively slower. Inevitably, the inference time of the model becomes longer with the introduction of SWTB architecture. However, the loss of time extrapolation is a necessary concession to the improvement of reconstruction accuracy.

**Figure 7:**
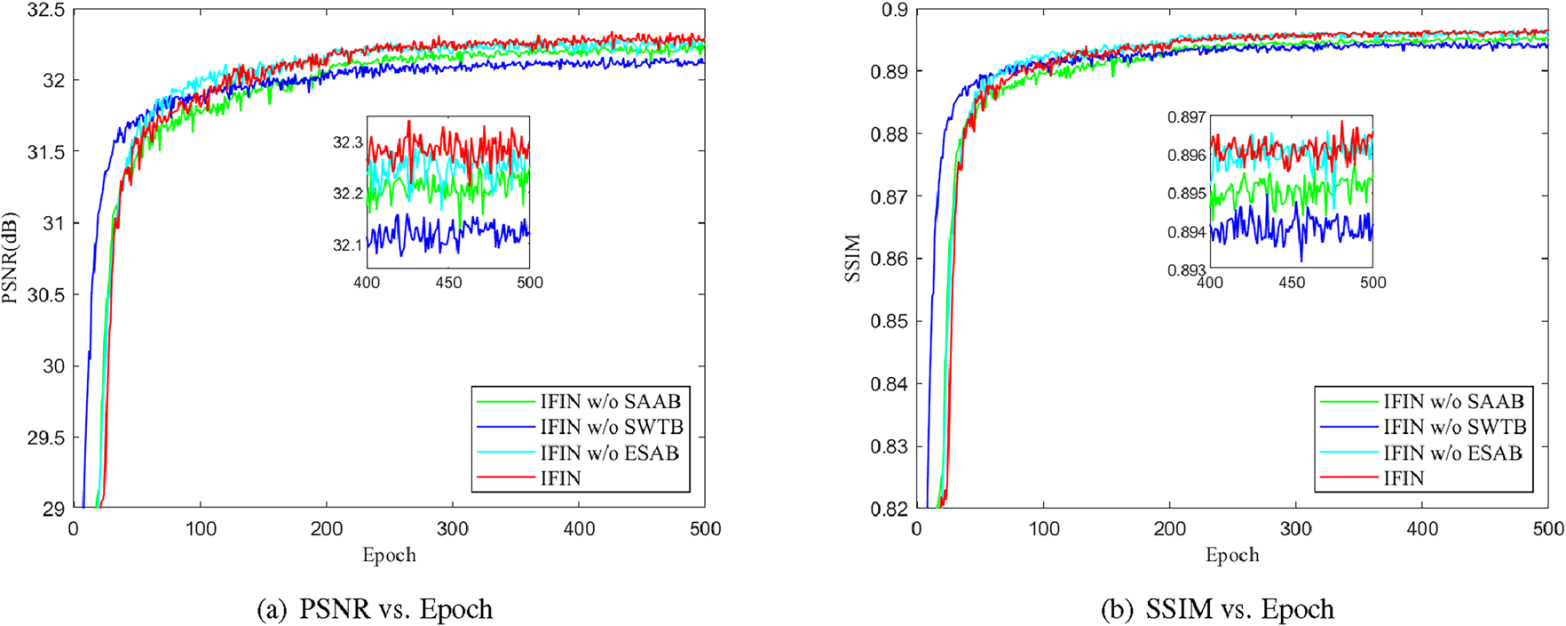
Convergence results of different models on Set5 for scale factor $\times$4.
3. Investigation of ESAB. As one of the key components of IFIN, the proposed ESAB effectively explores local structural and global dependence from SAAB and SWTB for better feature aggregation. It can be seen from Table 2 that IFIN augmented with ESAB attains improvements of 0.06dB in PSNR and 0.0010 in SSIM on Urban100, albeit with an increase in the number of model parameters by 8.2% and Multi-Adds by 20%. Additionally, we give visual heatmaps of IFIN with and without ESAB in Fig. 8, observing how ESAB acts on local and global responses. From Fig. 8a–e, high-frequency texture features present blurry and checkerboard artifacts in the absence of ESAB. On the contrary, detailed features of the image are clearer and more comprehensive after ESAB processing in Fig. 8f–j. Among them, the model not only focuses on the repeated small patterns but also emphasizes the sharp edge details. Combining all the improvements, SAAB, ESAB, and SWTB have exhibited great reasonableness and effectiveness.

**Figure 8:**
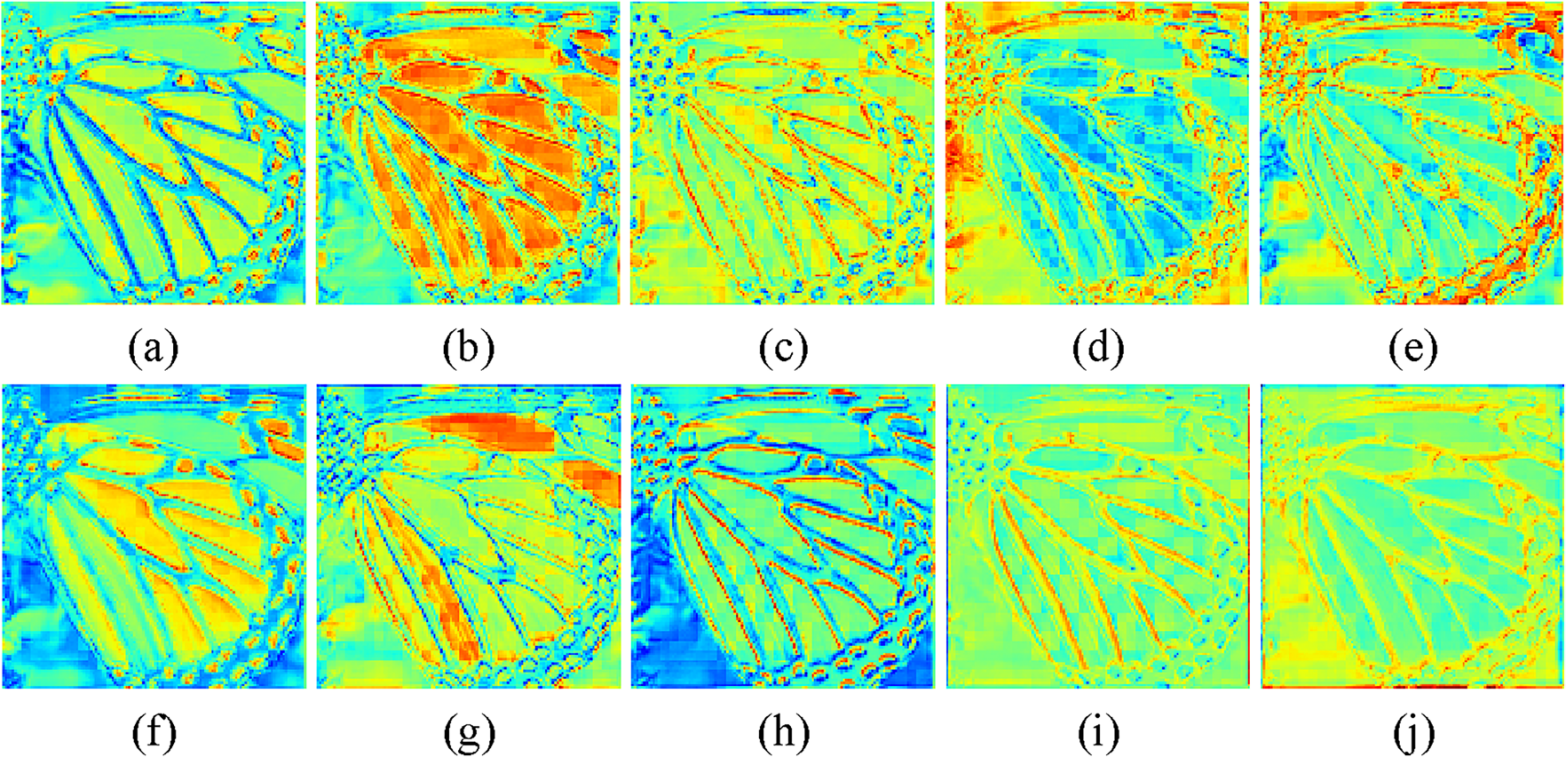
Visualized feature maps of IFIN with and without ESAB. (**a**–**e**) Heatmaps of IFIN without ESAB, (**f**–**j**) show heatmaps of IFIN with ESAB.
4. Investigation of IFAM. In Fig. 9, we illustrate visual features to analyze the interaction between the modules within the last IFAM. Based on the visualization, the output features of the three modules demonstrate minimal attention towards the low-frequency regions. Specifically, the output feature of SAAB focuses on texture structure details, such as lines and small patterns. In contrast, the output feature of SWTB shows an even distribution of activation values across the feature map. More importantly, the output characteristics of ESAB fuse global and local properties, resulting in a more pronounced representation of the target area and higher overall activation values. This observation suggests that the complementary fusion of local and global features facilitates the generation of additional high-frequency information, thereby aiding in the reconstruction of high-quality images.

**Figure 9:**
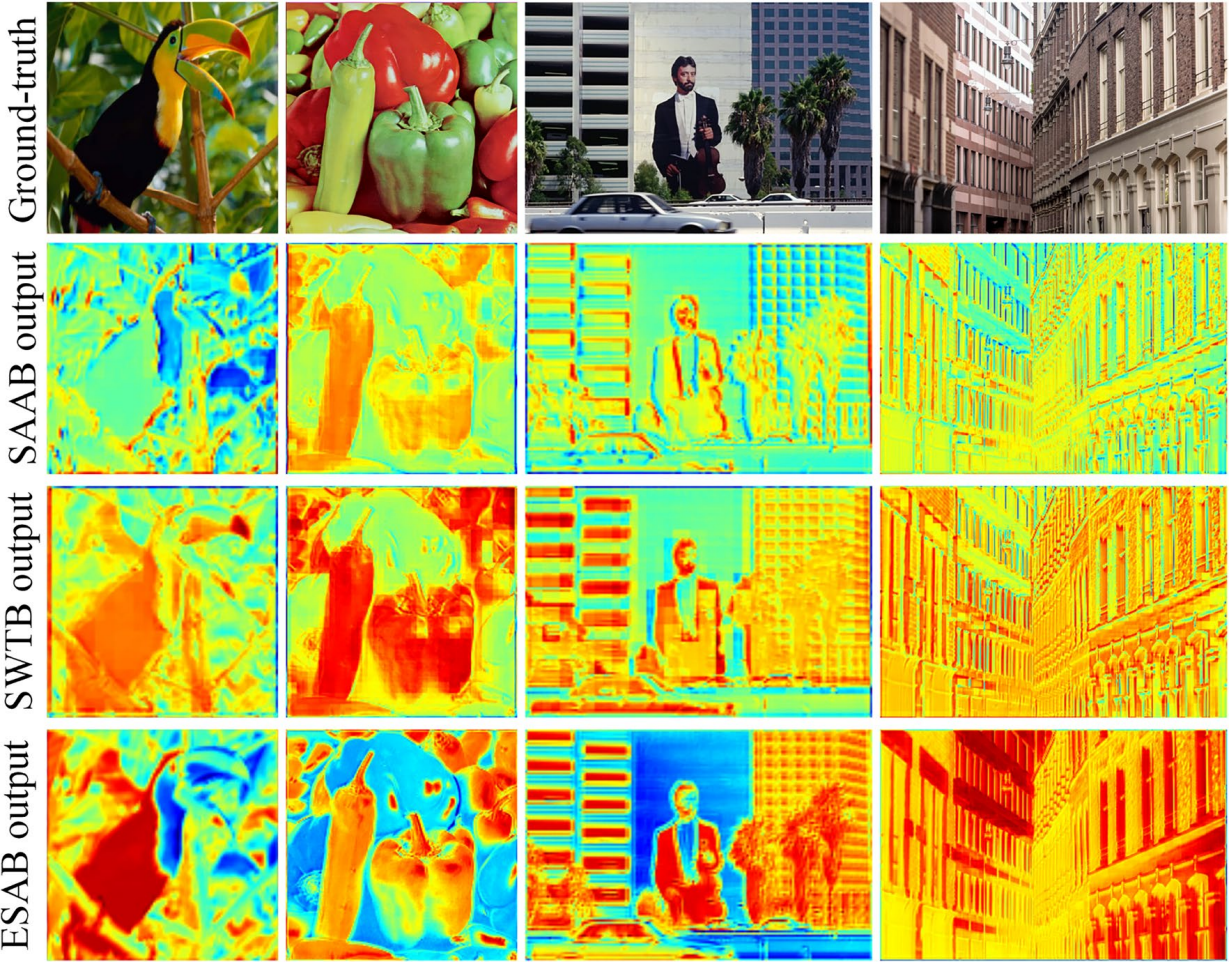
Average feature visualization inside the last IFAM.
5. Analysis of different numbers of IFAMs. To investigate the impact of model depth on network performance, we conduct analyses by varying the number of IFAM, setting *T* to 2, 3, 4, 5, and 6. As reported in Table 3, one can find that increasing the depth of the model by adding more IFAMs generally leads to better performance in terms of both PSNR and SSIM, but performance changes slowly when *T* exceeds 5. Meanwhile, we opt for *T* = 5 as the number of IFAMs to balance model performance against computational costs.

**Table 3:** Average PSNR and SSIM results on Set14 for scale factor $\times$4.

T	Params	Multi-adds	PSNR (dB)	SSIM
2	393K	99G	33.52	0.9160
3	586K	146G	33.57	0.9168
4	779K	195G	33.65	0.9177
5	972K	244G	33.73	0.9182
6	1165K	292G	33.76	0.9185

### Results with BI degradation

We make comparisons of the proposed IFIN-S and IFIN against existing SR approaches on the BI degradation model: SRCNN^1^, FSRCNN^38^, VDSR^15^, DRCN^5^, LapSRN^39^, DRRN^40^, MemNet^20^, CARN^6^, CBPN^41^, AWSRN-M^42^, OISR-RK2-s^43^, A2F-S^9^, LESRCNN^44^, SPBP-L^45^, RMUN^46^, FALSR^47^, WMRN^48^, LMAN-s^49^, MADNet-L1^50^, MSWSR^51^, Cross-SRN^52^, ACNet^29^, CRMBN^53^, DRSAN-48m^8^, FMEN^54^, AFAN^55^, ESRT^56^, LBNet^57^, CFGN^58^, and Ngswin^59^.

### Quantitative comparison

For a practical comparison that aligns with real-world application needs, we focus on selecting mainstream models that have a total network parameter count of less than 2000K. To enhance the comprehensibility of the comparison, we utilize Multi-Adds calculated by recovering a 1280 $\times$ 720 (720P) HR image. According to the results presented in Tables 4, 5 and 6, the proposed IFIN-S and IFIN exhibit competitive or superior PSNR and SSIM at different scales compared to popular SR networks. Compared to CNN-based methods, IFIN-S and IFIN exhibit better reconstruction performance at similar computational complexity. Note that our IFIN-S shows comparable results to CFGN, Cross-SRN, and FMFN, which suffer from more parameters and computations than ours. And IFIN-S achieves competitive results with ESRT while requiring less model capacity. Additionally, IFIN stands out by producing promising SR results with modest network parameters and Multi-Adds, even when compared to recently proposed Transformer-based methods. For example, in Table 6, IFIN has 0.19 dB higher PSNR and 0.0016 higher SSIM on Urban100 for $\times$4 over Ngswin. Although the Multi-Adds of IFIN are higher than that of Ngswin, the judicious increase in Multi-Adds is a necessary trade-off for improving accuracy. In essence, the superiority of our proposed methods is even more remarkable in reconstructing large-scale factors. This phenomenon can be attributed to the fact that LR images contain fewer pixel values at larger scale factors, necessitating the extraction of richer features to accurately restore HR images. Our proposed models, which implement both local and global strategies, exhibit strong representational capabilities. This strategy enables our network to capture intricate details more effectively, thereby enhancing performance significantly in SR tasks.

**Table 4:** Quantitative comparison on benchmark datasets for scale factor $\times$2.

Type	Model	Params	Multi-Adds	Set5	Set14	B100	Urban100	Manga109
				PSNR(dB)/SSIM	PSNR(dB)/SSIM	PSNR(dB)/SSIM	PSNR(dB)/SSIM	PSNR(dB)/SSIM
CNN-based	SRCNN^1^	57K	52.7G	36.66/0.9542	32.42/0.9063	31.36/0.8879	29.50/0.8946	35.74/0.9661
	FSRCNN^38^	12K	6G	37.00/0.9558	32.63/0.9088	31.53/0.8920	29.88/0.9020	36.67/0.9694
	VDSR^15^	665K	612.6G	37.53/0.9587	33.03/0.9124	31.90/0.8960	30.76/0.9140	37.22/0.9729
	DRCN^5^	1774K	17974G	37.63/0.9588	33.04/0.9118	31.85/0.8942	30.75/0.9133	37.63/0.9723
	LapSRN^39^	813K	29.9G	37.52/0.9590	33.08/0.9130	31.80/0.8950	30.41/0.9100	37.27/0.9740
	DRRN^40^	297K	6797G	37.74/0.9591	33.23/0.9136	32.05/0.8973	31.23/0.9188	37.92/0.9760
	CARN^6^	1592K	222.8G	37.76/0.9590	33.52/0.9166	32.09/0.8978	31.92/0.9256	-
	CBPN^41^	1036K	240.7G	37.90/0.9590	33.60/0.9171	32.17/0.8989	32.14/0.9279	-
	AWSRN-M^42^	1063K	244.1G	38.04/0.9605	33.66/0.9181	32.21/0.9000	32.23/0.9294	38.66/0.9772
	OISR-RK2-s^43^	1372 K	316.2G	37.98/0.9604	33.58/0.9172	32.18/0.8996	32.09/0.9281	-
	A2F-S^9^	320K	71.7G	37.79/0.9597	33.32/0.9152	31.99/0.8972	31.44/0.9211	38.11/0.9757
	LESRCNN^44^	516K	110.6G	37.65/0.9586	33.32/0.9148	31.95/0.8964	31.45/0.9206	-
	FALSR-A^47^	1021K	234.7G	37.82/0.9595	33.55/0.9168	32.12/0.8987	31.93/0.9256	-
	LMAN-s^49^	525K	121.2G	37.94/0.9603	33.49/0.9167	32.08/0.8984	31.85/0.9251	38.43/0.9765
	MSWSR^51^	1228 K	192.5 G	37.49/0.9583	33.23/0.9123	31.88/0.8929	31.14/0.9169	37.32/0.9733
	Cross-SRN^52^	1276K	292.1G	38.03/0.9606	33.62/0.9180	32.19/0.8997	32.28/0.9290	38.75/0.9773
	FMEN^54^	748K	172.0G	**38.10**/0.9609	33.75/0.9192	**32.26**/0.9007	32.41/0.9311	38.95/*0.9778*
	AFAN^55^	1208K	289.5G	38.06/0.9608	33.74/0.9198	32.20/0.9001	32.38/0.9305	38.79/0.9773
	ACNet^29^	1356K	501.5G	37.72/0.9588	33.41/0.9160	32.06/0.8978	31.79/0.9245	-
	CFGN^58^	601K	130.9G	38.03/0.9607	33.74/0.9197	32.23/0.9004	32.47/0.9316	38.96/**0.9777**
Tranformered-based	DRSAN-48m^8^	1188K	274.6G	*38.14/0.9611*	33.75/0.9188	32.25/*0.9010*	32.46/0.9317	-
	ESRT^56^	677K	271G	38.03/0.9600	33.75/0.9184	32.25/0.9001	*32.58*/**0.9318**	*39.12*/0.9774
	Ngswin^59^	998K	140.4G	38.05/**0.9610**	**33.79/0.9199**	*32.27*/**0.9008**	**32.53**/0.9324	**38.97/0.9777**
	IFIN-S(ours)	451K	110.6G	38.00/0.9606	33.66/0.9181	32.18/0.8996	32.14/0.9284	38.70/0.9771
	IFIN(ours)	972K	243.8G	**38.10/0.9610**	*33.90/0.9206*	**32.26**/0.9007	32.45/0.9314	38.95/**0.9777**

Italics and bold respectively denote the best and second-best performance.

**Table 5:** Quantitative comparison on benchmark datasets for scale factor $\times$3.

Type	Model	Params	Multi-Adds	Set5	Set14	B100	Urban100	Manga109
				PSNR(dB)/SSIM	PSNR(dB)/SSIM	PSNR(dB)/SSIM	PSNR(dB)/SSIM	PSNR(dB)/SSIM
CNN-based	SRCNN^1^	57K	52.7G	32.75/0.9090	29.28/0.8209	28.41/0.7863	26.24/0.7989	30.59/0.9107
	FSRCNN^38^	12K	5G	33.16/0.9140	29.43/0.8242	28.53/0.7910	26.43/0.8080	30.98/0.9212
	VDSR^15^	665K	612.6G	33.66/0.9213	29.77/0.8314	28.82/0.7976	27.14/0.8279	32.01/0.9310
	DRCN^5^	1774K	17974G	33.82/0.9226	29.76/0.8311	28.80/0.7963	27.15/0.8276	32.31/0.9328
	DRRN^40^	297K	6797G	34.03/0.9244	29.96/0.8349	28.95/0.8004	27.53/0.8378	32.74/0.9390
	MemNet^20^	677K	2662.4G	34.09/0.9248	30.00/0.8350	28.96/0.8001	27.56/0.8376	-
	CARN^6^	1592K	118.8G	34.29/0.9255	30.29/0.8407	29.06/0.8034	28.06/0.8493	-
	AWSRN-S^42^	477K	48.6G	34.02/0.9240	30.09/0.8376	28.92/0.8009	27.57/0.8391	32.82/0.9393
	AWSRN-M^42^	1143K	116.6G	34.42/0.9275	30.32/0.8419	29.13/0.8059	28.26/0.8545	33.64/0.9450
	OISR-RK2-s^43^	1557K	160.1 G	34.43/0.9273	30.33/0.8420	29.10/0.8053	28.20/0.8534	-
	A2F-S^9^	324k	32.3G	34.06/0.9241	30.08/0.8370	28.92/0.8006	27.57/0.8392	32.86/0.9394
	LESRCNN^44^	516K	49.1G	33.93/0.9231	30.12/0.8380	28.91/0.8005	27.70/0.8415	-
	WMRN^48^	556K	57G	34.11/0.9251	30.17/0.8390	28.98/0.8021	27.80/0.8448	33.07/0.9413
	LMAN-s^49^	709K	73.5G	34.31/0.9265	30.24/0.8397	29.02/0.8030	28.02/0.8487	33.42/0.9433
	Cross-SRN^52^	1285K	130.5G	34.43/0.9275	30.33/0.8417	29.09/0.8050	28.23/0.8535	33.65/0.9448
	ACNet^29^	1541K	369G	34.14/0.9247	30.19/0.8398	28.98/0.8023	27.97/0.8482	-
	FMEN^54^	757K	77.2G	34.45/0.9275	30.40/0.8435	29.17/0.8063	28.33/0.8562	33.86/0.9462
	AFAN^55^	1208K	143.1G	34.46/0.9271	30.38/0.8436	29.11/0.8064	28.31/0.8556	33.61/0.9451
	CFGN^58^	609K	59.0G	34.41/0.9274	30.44/0.8443	29.16/0.8066	28.37/0.8575	33.81/0.9459
Tranformered-based	DRSAN-48m^8^	1292K	133.4G	34.59/0.9286	30.42/0.8443	29.18/0.8079	28.52/0.8593	-
	ESRT^56^	770K	135G	34.42/0.9268	30.43/0.8433	29.15/0.8063	28.46/0.8574	33.95/0.9455
	LBNet^57^	736K	68.4G	34.47/0.9277	30.38/0.8417	29.13/0.8061	28.42/0.8559	33.82/0.9460
	Ngswin^59^	1007K	66.6G	34.52/0.9282	30.53/ 0.8456	29.19/0.8078	28.52/0.8603	33.89/0.9470
	IFIN-S (ours)	459K	51.0G	34.45/0.9278	30.47/0.8442	29.13/0.8064	28.32/0.8560	33.78/0.9460
	IFIN (ours)	980K	107.0G	34.58/0.9287	30.55/0.8463	29.21/0.8082	28.57/0.8608	34.14/0.9479

**Table 6:** Quantitative comparison on benchmark datasets for scale factor $\times$4.

Type	Model	Params	Multi-Adds	Set5	Set14	B100	Urban100	Manga109
				PSNR(dB)/SSIM	PSNR(dB)/SSIM	PSNR(dB)/SSIM	PSNR(dB)/SSIM	PSNR(dB)/SSIM
CNN-based	SRCNN^1^	57K	52.7G	30.48/0.8628	27.49/0.7503	26.90/0.7101	24.52/0.7221	27.66/0.8505
	FSRCNN^38^	12K	4.6G	30.71/0.8657	27.59/0.7535	26.98/0.7150	24.62/0.7280	27.90/0.8517
	VDSR^15^	665K	612.6G	31.35/0.8838	28.01/0.7674	27.29/0.7251	25.18/0.7524	28.83/0.8809
	DRCN^5^	1774K	17974G	31.53/0.8854	28.02/0.7670	27.23/0.7233	25.14/0.7510	28.98/0.8816
	LapSRN^39^	813K	149.4G	31.54/0.8850	28.19/0.7720	27.32/0.7280	25.21/0.7560	29.09/0.8845
	DRRN^40^	297K	6797G	31.68/0.8888	28.21/0.7720	27.38/0.7284	25.44/0.7638	29.46/0.8960
	MemNet^20^	677K	2662.4G	31.74/0.8893	28.26/0.7723	27.40/0.7281	25.50/0.7630	-
	CARN^6^	1592K	90.9G	32.13/0.8937	28.60/0.7806	27.58/0.7349	26.07/0.7837	-
	CBPN^41^	1197K	97.9G	32.21/0.8944	28.63/0.7813	27.58/0.7356	26.14/0.7869	-
	AWSRN-M^42^	1254K	72.0G	32.21/0.8954	28.65/0.7832	27.60/0.7368	26.15/0.7884	30.56/0.9093
	OISR-RK2-s^43^	1520 K	114.2 G	32.21/0.8950	28.63/0.7822	27.58/0.7364	26.14/0.7874	-
	A2F-S^9^	331k	18.6G	31.87/0.8900	28.36/0.7760	27.41/0.7305	25.58/0.7685	29.77/0.8987
	LESRCNN^44^	516K	28.6G	31.88/0.8903	28.44/0.7772	27.45/0.7313	25.77/0.7732	-
	WMRN^48^	536K	45.7G	32.00/0.8925	28.47/0.7786	27.49/0.7328	25.89/0.7789	30.11/0.9040
	LMAN-s^49^	672K	65.5G	32.12/0.8939	28.53/0.7798	27.51/0.7340	25.96/0.7813	30.30/0.9062
	MSWSR^51^	1228 K	101.7G	32.01/0.8914	28.47/0.7776	27.48/0.7311	25.78/0.7744	30.01/0.8999
	Cross-SRN^52^	1296K	74.2G	32.24/0.8954	28.59/0.7817	27.58/0.7364	26.16/0.7881	30.53/0.9081
	ACNet^29^	1784K	347.9G	31.83/0.8903	28.46/0.7788	27.48/0.7326	25.93/0.7798	-
	CRMBN^53^	495K	-	32.07/0.8923	28.50/0.7783	27.50/0.7327	25.83/0.7771	-
	FMEN^54^	769K	44.2G	32.24/0.8955	28.70/0.7839	27.63/0.7379	26.28/0.7908	30.70/0.9107
	AFAN^55^	1226K	90.1G	32.30/0.8951	28.66/0.7838	27.61/0.7383	26.27/0.7913	30.63/0.9109
	CFGN^58^	621K	33.9G	32.32/0.8957	28.67/0.7832	27.60/0.7377	26.22/0.7916	30.63/0.9101
Tranformered-based	DRSAN-48m^8^	1271K	88.7G	32.34/0.8960	28.65/0.7841	27.63/0.7390	26.33/0.7936	-
	ESRT^56^	751K	67.7G	32.19/0.8947	28.69/0.7833	27.69/0.7379	26.39/0.7962	30.75/0.9100
	LBNet^57^	742K	38.9G	32.29/0.8960	28.68/0.7832	27.62/0.7382	26.27/0.7906	30.76/0.9111
	Ngswin^59^	1019K	36.4G	32.33/0.8963	28.78/0.7859	27.66/0.7396	26.45/0.7963	30.80/0.9128
	IFIN-S(ours)	470K	31.6G	32.27/0.8958	28.68/0.7834	27.62/0.7381	26.17/0.7890	30.64/0.9106
	IFIN(ours)	991K	64.6G	32.47/0.8988	28.80/0.7871	27.70/0.7414	26.64/0.7979	31.06/0.9156

### Qualitative comparison

**Figure 10:**
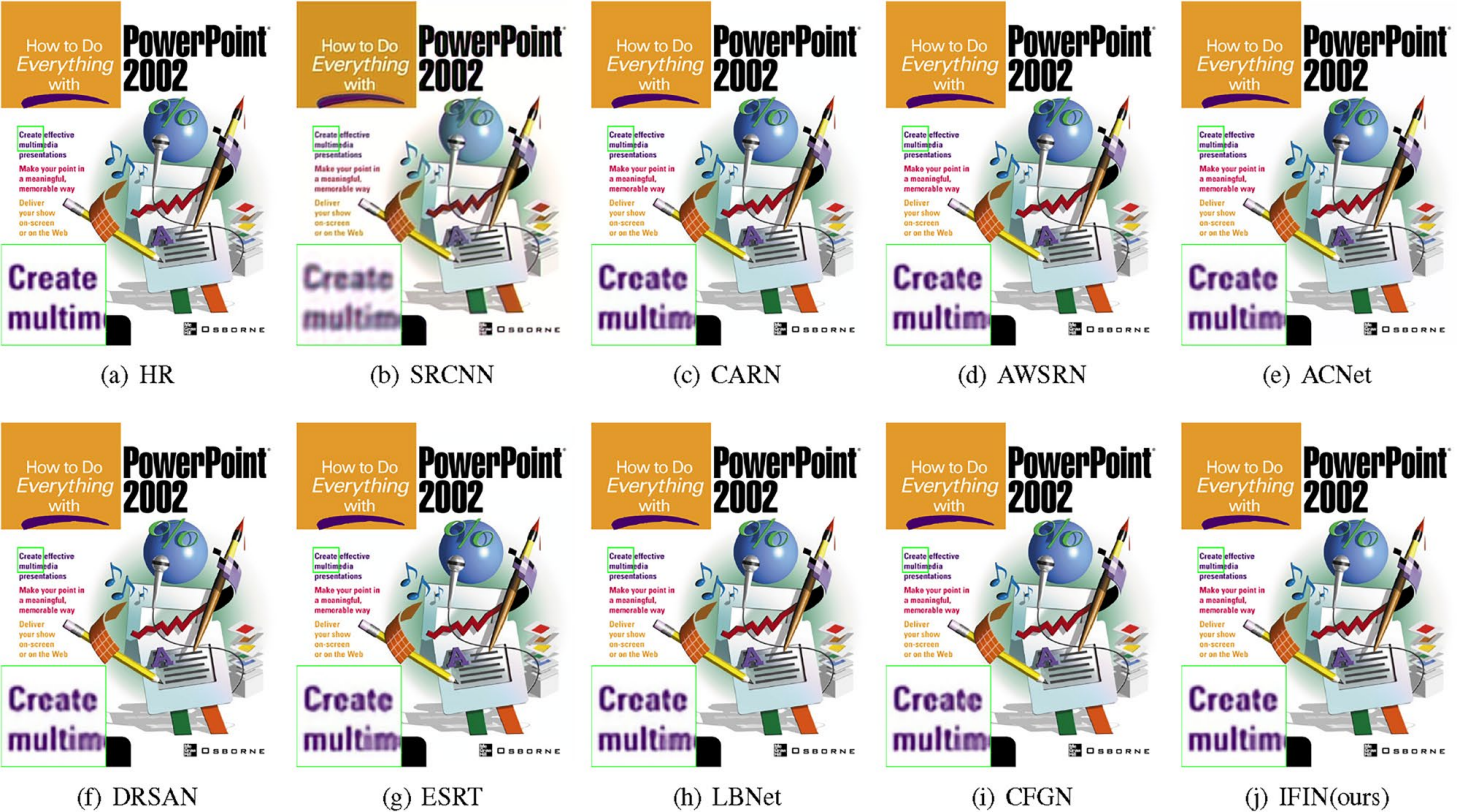
Qualitative comparison of popular networks on Set14 for scale factor $\times$2.

**Figure 11:**
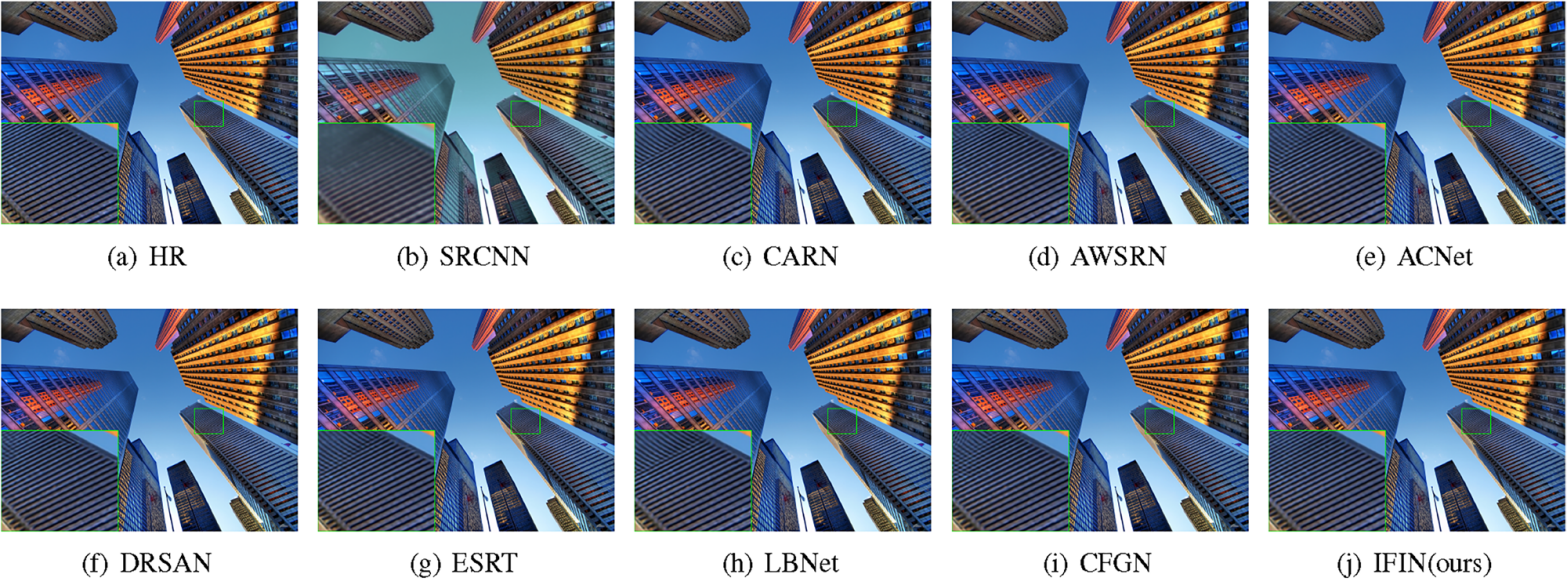
Qualitative comparison of popular networks on Urban100 for scale factor $\times$2.

**Figure 12:**
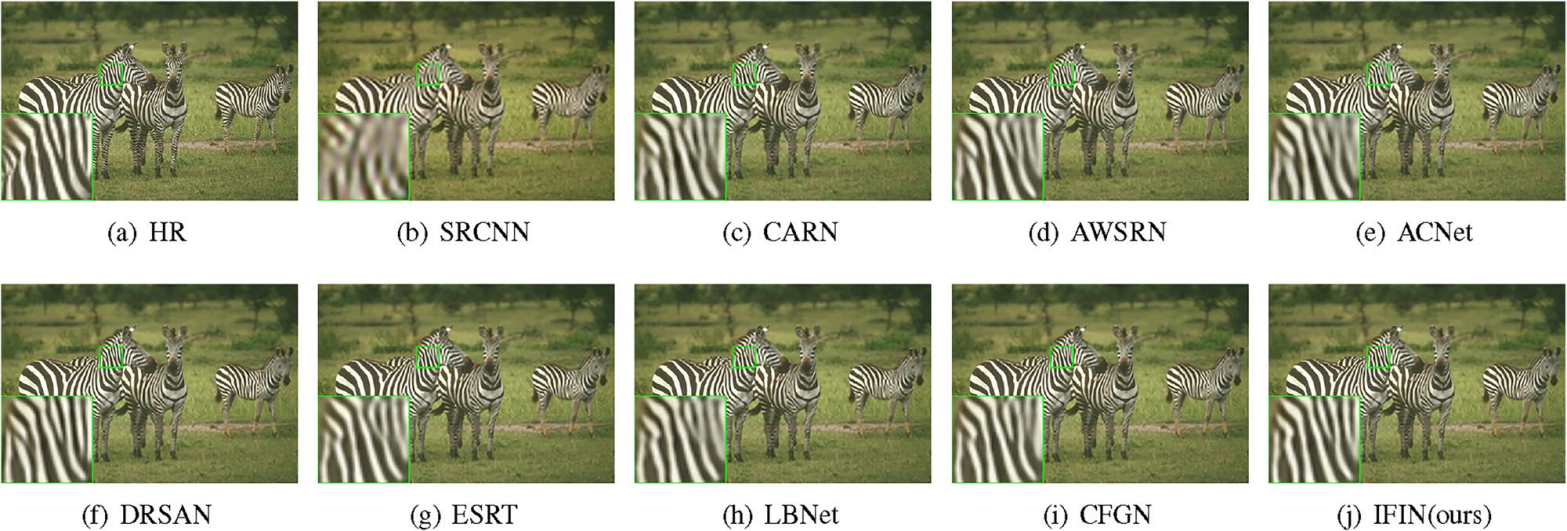
Qualitative comparison of popular networks on B100 for scale factor $\times$3.

**Figure 13:**
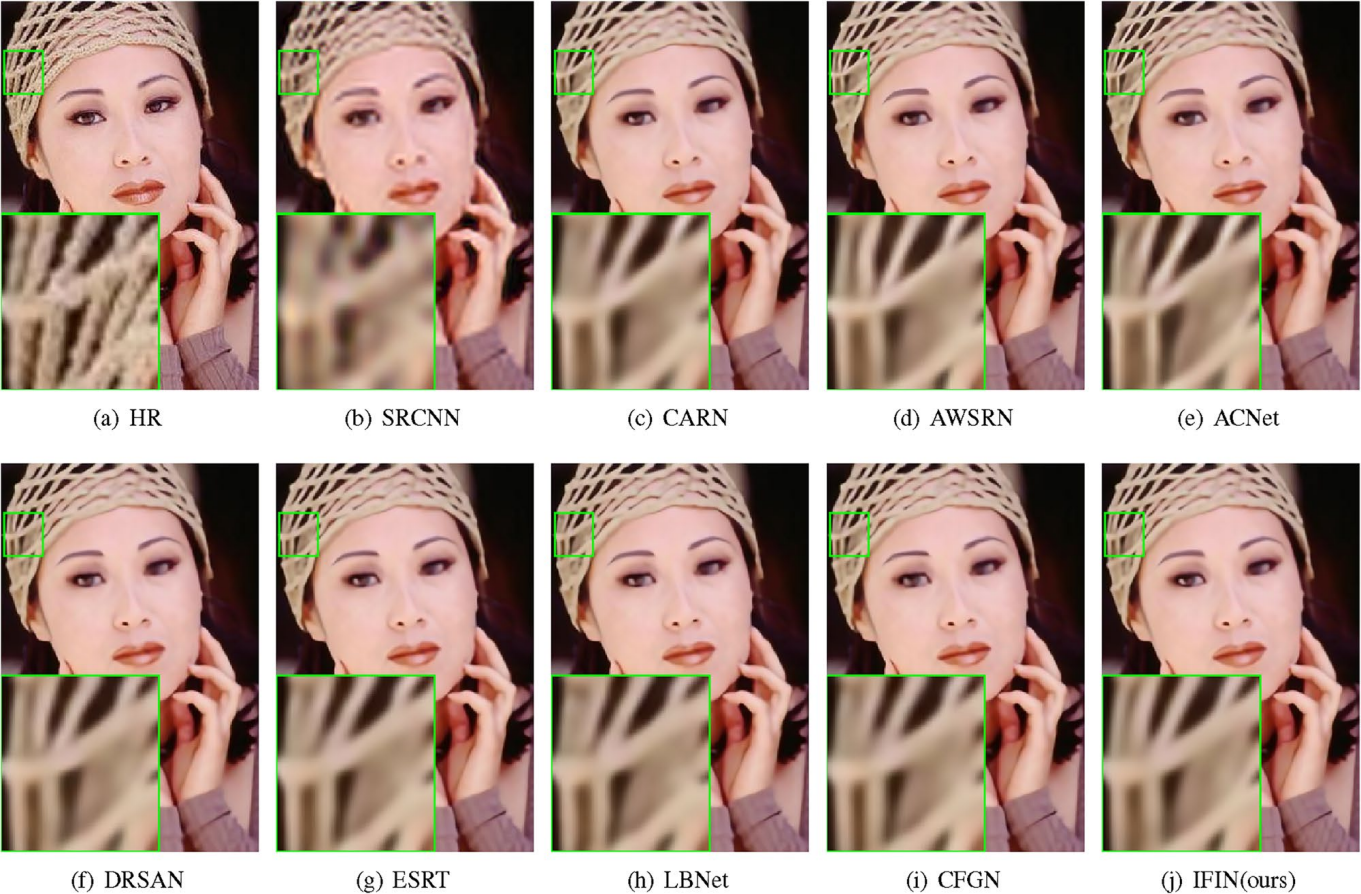
Qualitative comparison of popular networks on Set5 for scale factor $\times$4.

**Figure 14:**
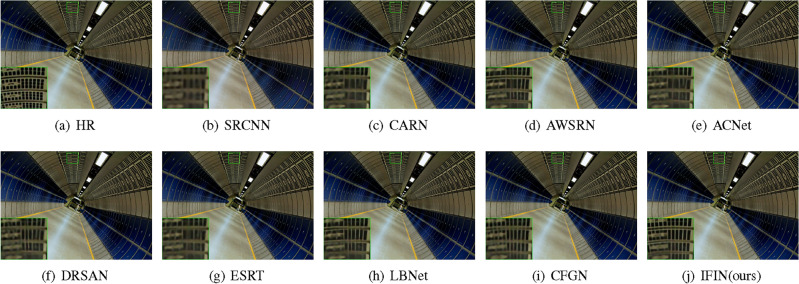
Qualitative comparison of popular networks on Urban100 for scale factor $\times$4.

We offer visual comparisons on selected portions of benchmark datasets, as depicted in Figs. 10, 11, 12, 13 and 14. Our IFIN exhibits superior restoration of stripes and line patterns, demonstrating finer and more accurate super-resolved images. As Fig. 10 depicts, our IFIN produces sharper details, which are close to HR image. In Figs. 12 and 13, ESRT, LBNet, and CFGN can yield stripe characteristics but with visible blurring. For Figs. 11 and 14, which are rich in stripe information, the comparison methods show severe distortions and deformations. Conversely, our IFIN effectively mitigates these issues, thereby recovering finer and more accurate detail information. This efficacy stems from IFIN’s specialization in capturing minute textures and extracting high-frequency cues, resulting in clearer and more precise image restorations.

### Results with BD and DN degradations

In this section, we conduct SR experiments on BD and DN degradation models to display the effectiveness and robustness of the proposed IFIN further. In this comparison, IFIN is evaluated against some popular SR methods, containing SRCNN^1^, FSRCNN^38^, VDSR^15^, IRCNN_G^60^, IRCNN_C^60^, SRMDNF^61^, RDN^16^, and AFAN^55^. According to the results presented in Table 7, our IFIN achieves optimal and suboptimal reconstruction results. Note that our IFIN outperforms RDN on the DN degradation model, which suffers from more parameters and operations than ours. The number of parameters and operations in IFIN are around 4.5% (0.98M vs. 22M) and 4.7% (107G vs. 2282G) of RDN, respectively. In addition, IFIN is superior to AFAN because it excels in exploring local and global priors, which considerably boosts the network’s discriminative ability. All experiments indicate that our IFIN strikes an advantageous trade-off between model capacity and reconstruction accuracy when compared to the state-of-the-art SR methods.

**Table 7:** Quantitative comparison on benchmark datasets for scale factor $\times$3.

Methods	Model	Set5	Set14	B100	Urban100	Manga109
		PSNR(dB)/SSIM	PSNR(dB)/SSIM	PSNR(dB)/SSIM	PSNR(dB)/SSIM	PSNR(dB)/SSIM
Bicubic	BD	28.34/0.8161	26.12/0.7106	26.02/0.6733	23.20/0.6661	25.46/0.8149
	DN	24.14/0.5445	23.14/0.4828	22.94/0.4461	21.63/0.4701	23.01/0.5381
SRCNN^1^	BD	31.75/0.8899	28.64/0.7997	27.33/0.7500	25.19/0.7591	29.47/0.8924
	DN	27.04/0.7638	25.56/0.6592	25.45/0.6198	23.59/0.6580	23.75/0.7148
FSRCNN^38^	BD	26.58/0.8224	24.86/0.7246	24.15/0.6728	22.95/0.6836	23.04/0.7927
	DN	24.28/0.7124	23.25/0.5956	23.95/0.5695	21.74/0.5724	22.39/0.7111
VDSR^15^	BD	33.29/0.9139	29.58/0.8259	28.61/0.7900	26.68/0.8019	31.06/0.9234
	DN	27.42/0.7372	25.60/0.6706	25.22/0.6271	23.33/0.6579	24.20/0.7525
IRCNN_G^60^	BD	33.38/0.9182	29.73/0.8292	28.65/0.7922	26.77/0.8154	31.15/0.9245
	DN	27.48/0.7925	25.92/0.6932	25.55/0.6481	23.93/0.6950	26.07/0.8253
IRCNN_C^60^	BD	33.17/0.9157	29.55/0.8271	28.49/0.7886	26.47/0.8081	31.13/0.9236
	DN	26.18/0.7430	24.68/0.6300	24.52/0.5850	22.63/0.6205	26.07/0.8253
SRMDNF^61^	BD	34.09/0.9242	30.11/0.8364	28.98/0.8009	27.50/0.8370	32.97/0.9391
	DN	27.74/0.8026	26.13/0.6974	25.64/0.6495	24.28/0.7092	26.72/0.8424
RDN^16^	BD	34.58/0.9280	30.53/0.8447	29.23/0.8079	28.46/0.8582	33.97/0.9465
	DN	28.47/0.8151	26.60/0.7101	25.93/0.6573	24.92/0.7364	28.00/0.8591
AFAN^55^	BD	34.48/0.9268	30.43/0.8430	29.14/0.8062	28.19/0.8521	33.77/0.9449
	DN	28.50/0.8156	26.58/0.7121	25.93/0.6618	24.84/0.7341	27.88/0.8581
IFIN(ours)	BD	34.59/0.9277	30.54/0.8442	29.22/0.8074	28.33/0.8547	34.16/0.9469
	DN	28.55/0.8180	26.64/0.7124	25.96/0.6615	24.94/0.7371	28.03/0.8607

### Results on real remote-sensing images

To further demonstrate the efficacy of our proposed methods, we test them on remote-sensing images of relatively low quality and spatial resolution. Following the methodologies established in works^62,63^, we use two test sets named RS-T1 and RS-T2 from the UC Merced dataset^64^. Both RS-1 and RS-2 contain 120 images and cover diverse scenes with complicated image patterns. We exploit existing remote-sensing SR methods for comparison, including SRCNN^1^, VDSR^15^, LGCNet^65^, LapSRN^39^, IDN^19^, LESRCNN^44^, CARN-M^6^, FENet^63^, FDENet^66^, and DRAN^67^. All the aforementioned methods are directly evaluated on remote sensing data utilizing pre-trained models provided by relevant workers. Meanwhile, these approaches are trained on the DIV2K dataset to ensure the fairness of comparison results.

**Figure 15:**
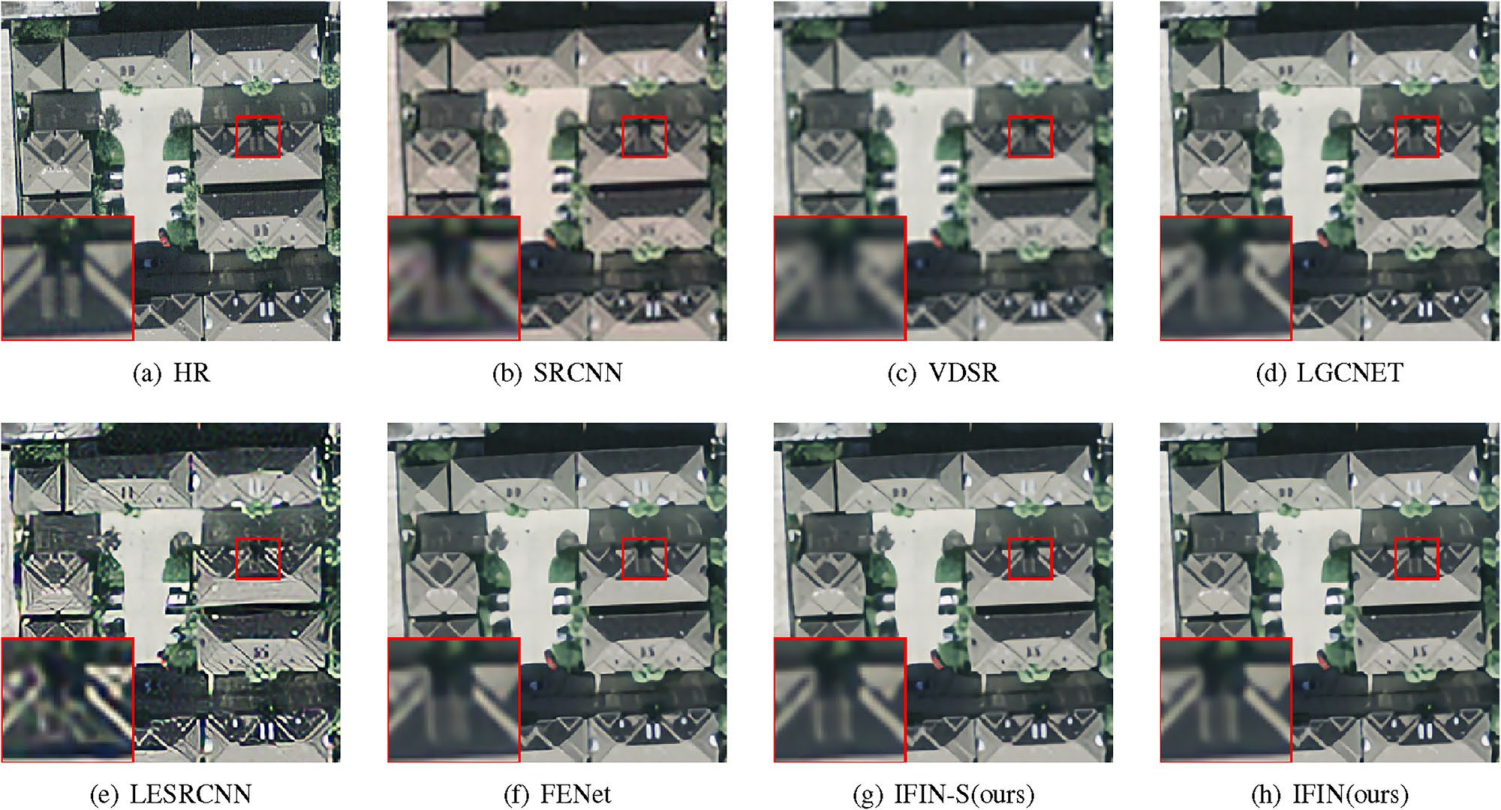
Qualitative comparison of popular networks on RS-T1 for scale factor $\times$3.

**Figure 16:**
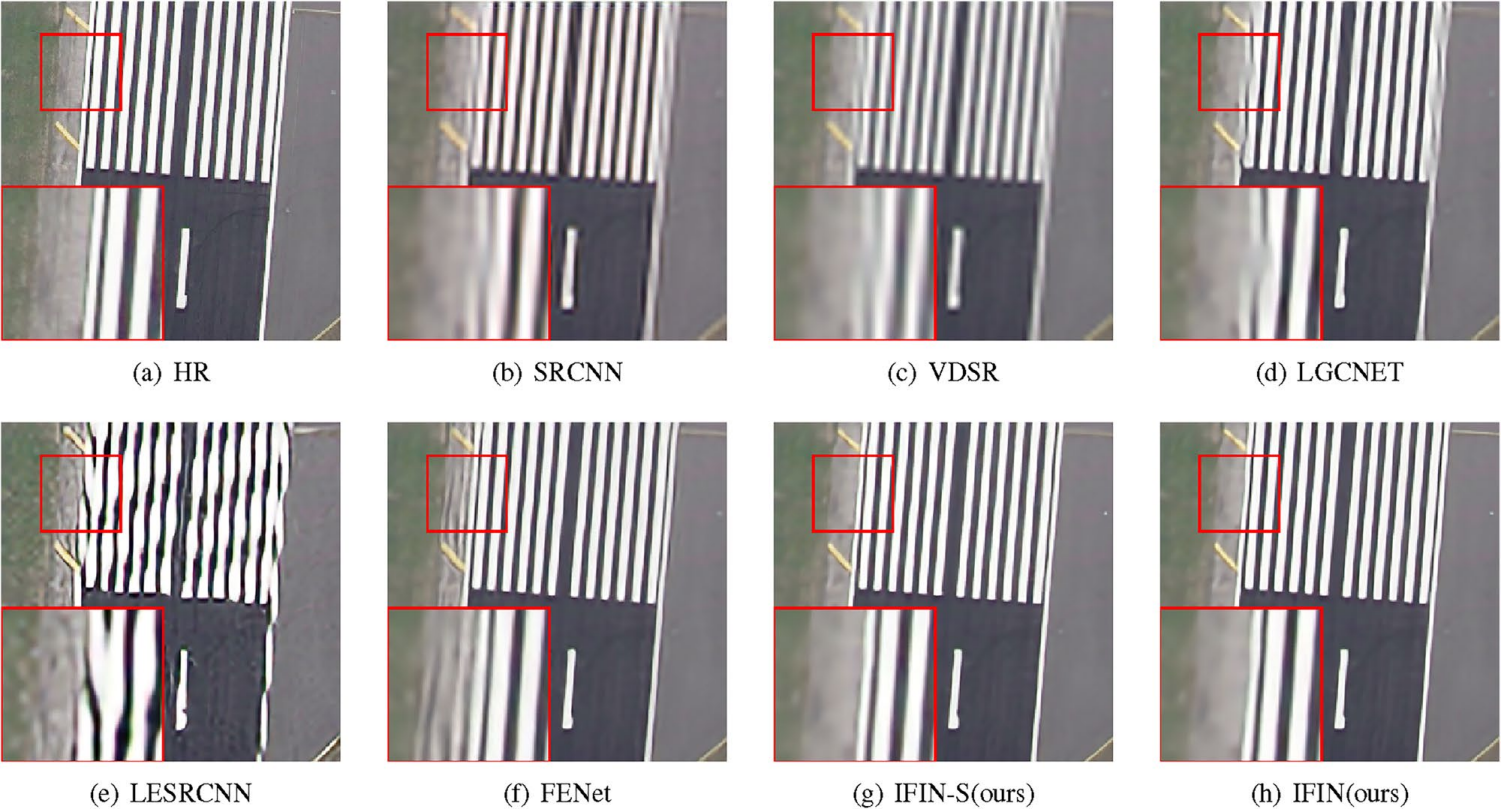
Qualitative comparison of popular networks on RS-T2 for scale factor $\times$4.

As presented in Table 8, it is noted that our IFIN obtains the best PSNR and SSIM scores, surpassing advanced remote sensing SR methods such as LGCNet, FeNet, DRAN, and FDENet. For instance, the proposed IFIN obtains 0.04-0.16 dB PSNR and 0.0004-0.0051 SSIM gains compared with lightweight DRAN. It is important to note that IFIN-S, which holds lower parameters than DRAN, attains competitive results on RS-T1 and RS-T2 datasets, exhibiting strong flexibility and stability. Figures 15 and  16 give visual comparisons of some methods. As we can see, our methods exhibit a better reconstruction effect than the comparison networks, particularly in terms of object outlines and texture details. In Fig. 16, LGCNET, LESRCNN, and FENet can not recover the stripe information, resulting in serious blurring, distortion, and artifacts respectively. Contrastively, our IFIN-S and IFIN with SAAB, SWTB, and ESAB can reconstruct details more clearly and accurately, visually consistent with the HR image.

**Table 8:** Quantitative comparison of remote-sensing datasets on RS-T1 and RS-T2.

Method	Scale	Params	RS-T1	RS-T2
			PSNR(dB)/SSIM	PSNR(dB)/SSIM
Bicubic	$\times$2	-	33.25/0.8934	30.64/0.8837
SRCNN^1^		57K	35.18/0.9243	32.87/0.9209
VDSR^15^		666K	35.85/0.9312	33.86/0.9312
LGCNet^65^		193K	35.65/0.9298	33.47/0.9281
LapSRN^39^		251K	35.69/0.9304	33.57/0.9286
IDN^19^		553K	36.13/0.9339	34.07/0.9329
LESRCNN^44^		626K	36.04/0.9328	34.00/0.9320
CARN-M^6^		412K	35.77/0.9314	33.84/0.9315
FeNet^63^		351K	36.23/0.9341	34.22/0.9337
FDENet^66^		480K	36.26/0.9346	34.28/0.9338
DRAN^67^		589K	36.38/0.9348	34.42/0.9357
IFIN-S(ours)		451K	36.38/0.9356	34.42/0.9352
IFIN(ours)		972K	36.42/0.9359	34.49/0.9361
Bicubic	$\times$3	-	29.73/0.7818	27.23/0.7697
SRCNN^1^		57K	30.95/0.8228	28.59/0.8180
VDSR^15^		666K	31.55/0.8352	29.40/0.8391
LGCNet^65^		193K	31.30/0.8314	29.03/0.8312
LapSRN^39^		290K	31.47/0.8338	29.22/0.8352
IDN^19^		553K	31.73/0.8430	29.59/0.8450
LESRCNN^44^		810K	31.68/0.8398	29.65/0.8444
CARN-M^6^		412K	31.72/0.8426	29.62/0.8452
FeNet^63^		357K	31.89/0.8432	29.80/0.8481
FDENet^66^		488K	31.98/0.8488	29.88/0.8489
DRAN^67^		589K	32.08/0.8470	30.05/0.8537
IFIN-S (ours)		470K	32.04/0.8448	30.03/0.8535
IFIN (ours)		991K	32.18/0.8506	32.18/0.8560
Bicubic	$\times$4	-	27.91/0.6968	25.40/0.6770
SRCNN^1^		57K	28.87/0.7382	26.46/0.7296
VDSR^15^		666K	29.33/0.7546	27.03/0.7525
LGCNet^65^		193K	29.13/0.7481	26.76/0.7426
LapSRN^39^		543K	29.51/0.7614	27.24/0.7600
IDN^19^		553K	29.56/0.7623	27.31/0.7627
LESRCNN^44^		774K	29.62/0.7625	27.41/0.7646
CARN-M^6^		412K	29.57/0.7624	27.37/0.7647
FeNet^63^		366K	29.70/0.7688	27.45/0.7672
FDENet^66^		501K	29.72/0.7658	27.54/0.7697
DRAN^67^		589K	29.85/0.7710	27.67/0.7758
IFIN-S(ours)		470K	29.84/0.7724	27.68/0.7763
IFIN(ours)		991K	29.92/0.7736	27.83/0.7809

### Model complexity

As we recognize the importance of model parameters and computational operations in designing lightweight methods, time consumption emerges as a critical metric for assessing the suitability of these methods for real-time applications. To this end, we perform time testing on some representative SR approaches exploiting the same device with an NVIDIA GeForce RTX 3060 GPU. Notably, we assess the time consumption of each method four times and subsequently compute the average score as the final result.

We depict the model parameters, Multi-Adds, PSNR/SSIM, and time consumption on B100 dataset in Table 9. Compared to CNN-based methods, the difference in performance and time cost between IFIN-S and AWSRN-M is not significant, but the computational complexity of IFIN-S is half that of AWSRN-M. Additionally, the time inference of CFGN is 24.7% longer than that of IFIN-S. Crucially, our IFIN obtains the highest PSNR and SSIM scores while utilizing fewer model parameters and calculations. However, the inclusion of a self-attention mechanism in ESAB, which necessitates a greater number of multiplication operations, somewhat diminishes the computational efficiency of our method. Nevertheless, our IFIN-S and IFIN exhibit superior computational efficiency in comparison to Transformer-based SR approaches. For instance, the time cost associated with DRSAN-48m and Ngswin is 8 and 2.5 times greater than that of our IFIN, respectively. Consequently, it is reasonable to conclude that our proposed IFIN-S and IFIN present a beneficial balance among network complexity, reconstruction accuracy, and time consumption.

**Table 9:** Comparison of model complexity on B100 for scale factor $\times$3.

Type	Model	Params	Multi-adds	PSNR(dB)/SSIM	Time (s)
CNN-based	CARN	1592K	118.8G	29.06/0.8034	0.4623
	AWSRN-S	477K	48.6G	28.92/0.8009	0.8807
	AWSRN-M	1143K	116.6G	29.13/0.8059	1.7432
	OISR-RK2-s	1557 K	160.1 G	29.10/0.8053	0.5304
	A2F-S	324k	32.3G	28.92/0.8006	0.6651
	LESRCNN	516K	49.1G	28.91/0.8005	0.2911
	LMAN-s	709K	73.5G	29.02/0.8030	1.6537
	Cross-SRN	1285K	130.5G	29.09/0.8050	2.2345
	ACNet	1541K	369G	28.98/0.8023	0.6436
	FMEN	757K	77.2G	29.17/0.8063	0.4598
	AFAN	1208K	143.1G	29.11/0.8064	2.9098
	CFGN	609K	59.0G	29.16/0.8066	2.1632
Transformer-based	DRSAN-48m	1292K	133.4G	29.18/0.8079	25.9941
	ESRT	770K	135G	29.15/0.8063	2.4120
	LBNet	736K	68.4G	29.13/0.8061	3.2489
	Ngswin	1007K	66.6G	29.19/0.8078	8.0806
	IFIN-S(ours)	459K	51.0G	29.13/0.8064	1.6288
	IFIN(ours)	980K	107.0G	29.21/0.8082	3.1728

## Limitations

In this study, our proposed method demonstrates slower inference speeds compared to most CNN-based methods, primarily due to the higher computational complexity of the Transformer. Also, although the structure we designed achieves high performance, there is still a limitation that we employ a fixed upsampling strategy. In future work, we intend to focus on improving the inference speed of the network and designing adaptive upsampling strategies while ensuring reconstruction accuracy.

## Conclusion

In this study, an efficient and lightweight interactive feature aggregation network (IFIN) is devised for the image SR task. Specifically, we propose an interactive feature aggregation module (IFAM), which consists of three key components: a structure-aware attention block (SAAB), a Swin Transformer block (SWTB), and an enhanced spatial adaptive block (ESAB). The SAAB focuses on capturing local salient structural features using asymmetric convolution, aiding in the restoration of texture details. Additionally, it collaborates with the SWTB to integrate global information efficiently into the ESAB. The ESAB plays a crucial role in seamlessly fusing and complementing local and global characteristics, generating more expressive feature representations and reconstructing natural and realistic image details. Extensive experiments indicate that our IFIN-S and IFIN are superior with respect to model capacity and reconstruction performance, exceeding mainstream lightweight SR methods.

## Data Availability

All datasets are publicly available. Correspondence and requests for materials should be addressed to Li Wang.

## References

[1] Dong, C., Loy, C. C., He, K. & Tang, X. Image super-resolution using deep convolutional networks. *IEEE Trans. Pattern Anal. Mach. Intell.* **38**, 295–307. 10.1109/TPAMI.2015.2439281 (2016). 26761735 10.1109/TPAMI.2015.2439281

[2] Zhang, Y. *et al.* Image super-resolution using very deep residual channel attention networks. In *European Conference on Computer Vision (ECCV)*. 286–301. 10.1007/978-3-030-01234-2_18 (2018).

[3] Lim, B., Son, S., Kim, H., Nah, S. & Mu Lee, K. Enhanced deep residual networks for single image super-resolution. In *Proceedings of the IEEE Conference on Computer Vision and Pattern Recognition (CVPR) Workshops*. 136–144. arXiv:org/abs/1707.02921 (2017).

[4] Mei, Y., Fan, Y. & Zhou, Y. Image super-resolution with non-local sparse attention. In *2021 IEEE/CVF Conference on Computer Vision and Pattern Recognition (CVPR)*. 3516–3525. 10.1109/CVPR46437.2021.00352 (2021).

[5] Kim, J., Lee, J. K. & Lee, K. M. Deeply-recursive convolutional network for image super-resolution. In *2016 IEEE Conference on Computer Vision and Pattern Recognition (CVPR)*. 1637–1645. 10.1109/CVPR.2016.181 (2016).

[6] Ahn, N., Kang, B. & Sohn, K.-A. *Fast, Accurate, and Lightweight Super-Resolution with Cascading Residual Network*. 256–272. 10.1007/978-3-030-01249-6_16 (2018) (event-place: Munich, Germany).

[7] Luo, X. *et al.* LatticeNet: Towards lightweight image super-resolution with lattice block. In *European Conference on Computer Vision (ECCV)*. 272–289. 10.1007/978-3-030-58542-6_17 (2020).

[8] Park, K., Soh, J. W. & Cho, N. I. A dynamic residual self-attention network for lightweight single image super-resolution. *IEEE Trans. Multimed.* **25**, 907–918. 10.1109/TMM.2021.3134172 (2023).

[9] Wang, X. *et al.* Lightweight single-image super-resolution network with attentive auxiliary feature learning. In *Asian Conference on Computer Vision(ACCV)*. 268–285. 10.1007/978-3-030-69532-3_17 (2020).

[10] Liu, Z. *et al.* Swin transformer: Hierarchical vision transformer using shifted windows. In *2021 IEEE/CVF International Conference on Computer Vision (ICCV)*. 9992–10002. 10.1109/ICCV48922.2021.00986 (2021).

[11] Zhang, X., Zeng, H., Guo, S. & Zhang, L. Efficient long-range attention network for image super-resolution. In *European Conference on Computer Vision (ECCV)*. 649–667. 10.1007/978-3-031-19790-1_39 (2022).

[12] Fang, J., Lin, H., Chen, X. & Zeng, K. A hybrid network of CNN and transformer for lightweight image super-resolution. In *Proceedings of the IEEE/CVF Conference on Computer Vision and Pattern Recognition (CVPR) Workshops*. 1103–1112. 10.1109/CVPRW56347.2022.00119 (2022).

[13] Li, W. *et al.* Cross-receptive focused inference network for lightweight image super-resolution. arXiv:2207.02796 [cs.CV] 10.48550/ARXIV.2207.02796 (2022).

[14] Yoo, J. *et al.* Enriched CNN-transformer feature aggregation networks for super-resolution. In *2023 IEEE/CVF Winter Conference on Applications of Computer Vision (WACV)*. 4945–4954. 10.1109/WACV56688.2023.00493 (2023).

[15] Kim, J., Lee, J. K. & Lee, K. M. Accurate image super-resolution using very deep convolutional networks. In *Proceedings of the IEEE Conference on Computer Vision and Pattern Recognition (CVPR)*. 1646–1654. 10.1109/CVPR.2016.182 (2016).

[16] Zhang, Y., Tian, Y., Kong, Y., Zhong, B. & Fu, Y. Residual dense network for image super-resolution. In *2018 IEEE/CVF Conference on Computer Vision and Pattern Recognition*. 2472–2481. 10.1109/CVPR.2018.00262 (2018).

[17] Niu, B. Single. *et al.* 16th European Conference, Glasgow, UK, August 23–28, 2020. *Proceedings, Part XII*. 191–207. https://doi.org/10.1007/978-3-030-58610-2_12 (Springer, 2020) (event-place: Glasgow, United Kingdom).

[18] Nguyen, Q. H. & Beksi, W. J. Single image super-resolution via a dual interactive implicit neural network. In *2023 IEEE/CVF Winter Conference on Applications of Computer Vision (WACV)*. 4925–4934. 10.1109/WACV56688.2023.00491 (IEEE, 2023).

[19] Hui, Z., Wang, X. & Gao, X. Fast and accurate single image super-resolution via information distillation network. In *2018 IEEE/CVF Conference on Computer Vision and Pattern Recognition*. 723–731. 10.1109/CVPR.2018.00082 (2018).

[20] Tai, Y., Yang, J., Liu, X. & Xu, C. MemNet: A persistent memory network for image restoration. In *2017 IEEE International Conference on Computer Vision (ICCV)*. 4549–4557. 10.1109/ICCV.2017.486 (2017).

[21] Hu, Y., Li, J., Huang, Y. & Gao, X. Channel-wise and spatial feature modulation network for single image super-resolution. *IEEE Trans. Circuits Syst. Video Technol.* **30**, 3911–3927. 10.1109/TCSVT.2019.2915238 (2020).

[22] Zhao, H., Kong, X., He, J., Qiao, Y. & Dong, C. *Efficient Image Super-Resolution Using Pixel Attention*. 56–72. 10.1007/978-3-030-67070-2_3 (Springer, 2020).

[23] Dosovitskiy, A. *et al.**An Image is Worth 16 x 16 Words: Transformers for Image Recognition at Scale*. arXiv:2010.11929 (2020).

[24] Chen, H. *et al.* Pre-trained image processing transformer. In *2021 IEEE/CVF Conference on Computer Vision and Pattern Recognition (CVPR)*. 12294–12305. 10.1109/CVPR46437.2021.01212 (2021).

[25] Lu, Z. *et al.* Transformer for single image super-resolution. In *2022 IEEE/CVF Conference on Computer Vision and Pattern Recognition Workshops (CVPRW)*. 456–465. 10.1109/CVPRW56347.2022.00061 (2022).

[26] Cai, Q. *et al.* HIPA: Hierarchical patch transformer for single image super resolution. *IEEE Trans. Image Process.* **32**, 3226–3237. 10.1109/TIP.2023.3279977 (2023). 37256801 10.1109/TIP.2023.3279977

[27] Yoo, J. *et al.**Rich CNN-Transformer Feature Aggregation Networks for Super-Resolution*. arXiv:2203.0768210.48550/arXiv.2203.07682 (2022).

[28] Yu, J. *et al.**Wide Activation for Efficient and Accurate Image Super-Resolution*. arXiv:1808.08718 [cs.CV] 10.48550/arXiv.1808.08718 (2018).

[29] Tian, C., Xu, Y., Zuo, W., Lin, C.-W. & Zhang, D. Asymmetric CNN for image superresolution. *IEEE Trans. Syst. Man Cybern. Syst.* **52**, 3718–3730. 10.1109/TSMC.2021.3069265 (2022).

[30] Xu, M., Peng, Y., Zhang, Y., Jia, X. & Jia, S. AACNet: Asymmetric attention convolution network for hyperspectral image dehazing. *IEEE Trans. Geosci. Remote Sens.* **61**, 1–14. 10.1109/TGRS.2023.3321294 (2023).

[31] Liang, J. *et al.* SwinIR: Image restoration using swin transformer. In *2021 IEEE/CVF International Conference on Computer Vision Workshops (ICCVW)*. 1833–1844. 10.1109/ICCVW54120.2021.00210 (2021).

[32] Timofte, R., Agustsson, E., Gool, L. V., Yang, M. H. & Guo, Q. NTIRE 2017 challenge on single image super-resolution: Methods and results. In *IEEE Conference on Computer Vision and Pattern Recognition Workshops (CVPRW)*. 114–125 (2017).

[33] Bevilacqua, M., Roumy, A., Guillemot, C. & Morel, M.-l. A. Low-complexity single-image super-resolution based on nonnegative neighbor embedding. In *Proceedings of the 23rd British Machine Vision Conference (BMVC)*. 1–10. 10.5244/C.26.135 (British Machine Vision Association, 2012).

[34] Zeyde, R., Elad, M. & Protter, M. On single image scale-up using sparse-representations. In *International Conference on Curves and Surfaces*. 711–730. 10.1007/978-3-642-27413-8_47 (2010).

[35] Martin, D., Fowlkes, C., Tal, D. & Malik, J. A database of human segmented natural images and its application to evaluating segmentation algorithms and measuring ecological statistics. In *Proceedings Eighth IEEE International Conference on Computer Vision(ICCV)*. Vol. 2. 416–423. 10.1109/ICCV.2001.937655 (2001).

[36] Huang, J.-B., Singh, A. & Ahuja, N. Single image super-resolution from transformed self-exemplars. In *2015 IEEE Conference on Computer Vision and Pattern Recognition (CVPR)*. 5197–5206. 10.1109/CVPR.2015.7299156 (2015).

[37] Matsui, Y. *et al.* Sketch-based Manga retrieval using Manga109 dataset. *Multimed. Tools Appl.* **76**, 21811–21838. 10.1007/s11042-016-4020-z (2017).

[38] Chao, D., Chen, C. L. & Tang, X. Accelerating the super-resolution convolutional neural network. In *European Conference on Computer Vision (ECCV)*. 391–407. 10.1007/978-3-319-46475-6_25 (2016).

[39] Lai, W.-S., Huang, J.-B., Ahuja, N. & Yang, M.-H. Deep Laplacian pyramid networks for fast and accurate super-resolution. In *2017 IEEE Conference on Computer Vision and Pattern Recognition (CVPR)*. 5835–5843. 10.1109/CVPR.2017.618 (2017).

[40] Tai, Y., Yang, J. & Liu, X. Image super-resolution via deep recursive residual network. In *2017 IEEE Conference on Computer Vision and Pattern Recognition (CVPR)*. 2790–2798. 10.1109/CVPR.2017.298 (2017).

[41] Zhu, F. & Zhao, Q. Efficient single image super-resolution via hybrid residual feature learning with compact back-projection network. In *2019 IEEE/CVF International Conference on Computer Vision Workshop (ICCVW)*. 2453–2460. 10.1109/ICCVW.2019.00300 (2019).

[42] Wang, C., Li, Z. & Shi, J. *Lightweight Image Super-Resolution with Adaptive Weighted Learning Network*. arXiv:1904.0235810.48550/arXiv.1904.02358 (2019).

[43] He, X. *et al.* ODE-inspired network design for single image super-resolution. In *2019 IEEE/CVF Conference on Computer Vision and Pattern Recognition (CVPR)*. 1732–1741. 10.1109/CVPR.2019.00183 (2019).

[44] Tian, C. *et al.* Lightweight image super-resolution with enhanced CNN. *Knowl.-Based Syst.* **205**, 106235. 10.1016/j.knosys.2020.106235 (2020).

[45] Banerjee, S., Ozcinar, C., Rana, A., Smolic, A. & Manzke, M. *Sub-Pixel Back-Projection Network For Lightweight Single Image Super-Resolution*. arXiv:2008.0111610.48550/arXiv.2008.01116 (2020).

[46] Jiang, Z., Zhu, H., Lu, Y., Ju, G. & Men, A. Lightweight super-resolution using deep neural learning. *IEEE Trans. Broadcast.* **66**, 814–823. 10.1109/TBC.2020.2977513 (2020).

[47] Chu, X., Zhang, B., Ma, H., Xu, R. & Li, Q. Fast, accurate and lightweight super-resolution with neural architecture search. In *2020 25th International Conference on Pattern Recognition (ICPR)*. 59–64. 10.1109/ICPR48806.2021.9413080 (2021).

[48] Sun, L. *et al.* Lightweight image super-resolution via weighted multi-scale residual network. *IEEE/CAA J. Autom. Sin.* **8**, 1271–1280. 10.1109/JAS.2021.1004009 (2021).

[49] Wan, J., Yin, H., Liu, Z., Chong, A. & Liu, Y. Lightweight image super-resolution by multi-scale aggregation. *IEEE Trans. Broadcast.* **67**, 372–382 (2021).

[50] Lan, R. *et al.* MADNet: A fast and lightweight network for single-image super resolution. *IEEE Trans. Cybern.* **51**, 1443–1453. 10.1109/TCYB.2020.2970104 (2021). 32149667 10.1109/TCYB.2020.2970104

[51] Zhang, H., Xiao, J. & Jin, Z. Multi-scale image super-resolution via a single extendable deep network. *IEEE J. Sel. Top. Signal Process.* **15**, 253–263. 10.1109/JSTSP.2020.3045282 (2021).

[52] Liu, Y. *et al.* Cross-SRN: Structure-preserving super-resolution network with cross convolution. *IEEE Trans. Circuits Syst. Video Technol.* **32**, 4927–4939. 10.1109/TCSVT.2021.3138431 (2022).

[53] Wei, D. & Wang, Z. Channel rearrangement multi-branch network for image super-resolution. *Digit. Signal Process.* **120**, 103254. 10.1016/j.dsp.2021.103254 (2022).

[54] Du, Z. *et al.* Fast and memory-efficient network towards efficient image super-resolution. In *2022 IEEE/CVF Conference on Computer Vision and Pattern Recognition Workshops (CVPRW)*. 852–861. 10.1109/CVPRW56347.2022.00101 (2022).

[55] Wang, L., Li, K., Tang, J. & Liang, Y. Image super-resolution via lightweight attention-directed feature aggregation network. *ACM Trans. Multimedia Comput. Commun. Appl.* **19**10.1145/3546076 (2023) (Association for Computing Machinery).

[56] Lu, Z., Liu, H., Li, J. & Zhang, L. *Efficient Transformer for Single Image Super-Resolution*. arXiv:2108.1108410.48550/arXiv.2108.11084 (2021).

[57] Gao, G. *et al.**Lightweight Bimodal Network for Single-Image Super-Resolution via Symmetric CNN and Recursive Transformer*. 896–902. 10.24963/ijcai.2022/126 (2022).

[58] Dai, T. *et al.* CFGN: A lightweight context feature guided network for image super-resolution. In *IEEE Transactions on Emerging Topics in Computational Intelligence*. 1–11. 10.1109/TETCI.2023.3289618 (2023).

[59] Choi, H., Lee, J. & Yang, J. N-Gram in Swin transformers for efficient lightweight image super-resolution. In *2023 IEEE/CVF Conference on Computer Vision and Pattern Recognition (CVPR)*. 2071–2081. 10.1109/CVPR52729.2023.00206 (2023).

[60] Zhang, K., Zuo, W. & Zhang, L. Learning a single convolutional super-resolution network for multiple degradations. In *2018 IEEE/CVF Conference on Computer Vision and Pattern Recognition*. 3262–3271. 10.1109/CVPR.2018.00344 (2018).

[61] Zhang, K., Zuo, W., Gu, S. & Zhang, L. Learning deep CNN denoiser prior for image restoration. In *2017 IEEE Conference on Computer Vision and Pattern Recognition (CVPR)*. 2808–2817. 10.1109/CVPR.2017.300 (2017).

[62] Dong, X. *et al.* Remote sensing image super-resolution using novel dense-sampling networks. *IEEE Trans. Geosci. Remote Sens.* **59**, 1618–1633. 10.1109/TGRS.2020.2994253 (2021).

[63] Wang, Z. *et al.* FeNet: Feature enhancement network for lightweight remote-sensing image super-resolution. *IEEE Trans. Geosci. Remote Sens.* **60**, 1–12. 10.1109/TGRS.2022.3168787 (2022).

[64] Yang, Y. & Newsam, S. Bag-of-visual-words and spatial extensions for land-use classification. In *Proceedings of the 18th SIGSPATIAL International Conference on Advances in Geographic Information Systems*, GIS ’10. 270–279. 10.1145/1869790.1869829 (Association for Computing Machinery, 2010) (event-place: San Jose, California).

[65] Lei, S., Shi, Z. & Zou, Z. Super-resolution for remote sensing images via local-global combined network. *IEEE Geosci. Remote Sens. Lett.* **14**, 1243–1247. 10.1109/LGRS.2017.2704122 (2017).

[66] Gao, F. *et al.* A lightweight feature distillation and enhancement network for super-resolution remote sensing images. *Sensors*10.3390/s23083906 (2023). 37112247 10.3390/s23083906 PMC10147051

[67] Wang, Q., Wang, S., Chen, M. & Zhu, Y. DARN: Distance attention residual network for lightweight remote-sensing image superresolution. *IEEE J. Sel. Top. Appl. Earth Obs. Remote Sens.* **16**, 714–724. 10.1109/JSTARS.2022.3227509 (2023).

